# A Virus-Specific Immune Rheostat in the Immunome of Patients Recovering From Mild COVID-19

**DOI:** 10.3389/fimmu.2021.674279

**Published:** 2021-05-25

**Authors:** Joo Guan Yeo, Jing Yao Leong, Shi Huan Tay, Karen Donceras Nadua, Danielle E. Anderson, Amanda Jin Mei Lim, Xiang Wen Ng, Su Li Poh, Dianyan Guo, Katherine Nay Yaung, Pavanish Kumar, Martin Wasser, Sharifah Nur Hazirah, Nursyuhadah Sutamam, Camillus Jian Hui Chua, Martin Qui, Randy Foo, Akshamal Mihiranga Gamage, Kee Thai Yeo, Lakshmi Ramakrishna, Thaschawee Arkachaisri, Barnaby E. Young, David Chien Lye, Lin-Fa Wang, Chia Yin Chong, Natalie Woon Hui Tan, Jiahui Li, Kai-Qian Kam, Florent Ginhoux, Koh Cheng Thoon, Jerry Kok Yen Chan, Chee Fu Yung, Salvatore Albani

**Affiliations:** ^1^ Translational Immunology Institute, SingHealth Duke-NUS Academic Medical Centre, Singapore, Singapore; ^2^ Rheumatology and Immunology Service, Department of Pediatric Subspecialities, KK Women’s and Children’s Hospital, Singapore, Singapore; ^3^ Duke-NUS Medical School, Singapore, Singapore; ^4^ Yong Loo Lin School of Medicine, National University of Singapore (NUS), Singapore, Singapore; ^5^ Infectious Disease Service, Department of Pediatrics, KK Women’s and Children’s Hospital, Singapore, Singapore; ^6^ Department of Reproductive Medicine, KK Women’s and Children’s Hospital, Singapore, Singapore; ^7^ Department of Neonatology, KK Women’s and Children’s Hospital, Singapore, Singapore; ^8^ National Centre for Infectious Diseases, Singapore, Singapore; ^9^ Department of Infectious Diseases, Tan Tock Seng Hospital, Singapore, Singapore; ^10^ Lee Kong Chian School of Medicine, Singapore, Singapore; ^11^ Singapore Immunology Network (SIgN), Agency for Science, Technology and Research (ASTAR), Singapore, Singapore

**Keywords:** COVID-19, SARS-CoV-2, regulatory T cells, follicular helper T cells, mass cytometry

## Abstract

An accurate depiction of the convalescent COVID-19 immunome will help delineate the immunological milieu crucial for disease resolution and protection. Using mass cytometry, we characterized the immune architecture in patients recovering from mild COVID-19. We identified a virus-specific immune rheostat composed of an effector T (T_eff_) cell recall response that is balanced by the enrichment of a highly specialized regulatory T (T_reg_) cell subset. Both components were reactive against a peptide pool covering the receptor binding domain (RBD) of the SARS-CoV-2 spike glycoprotein. We also observed expansion of IFNγ^+^ memory CD4^+^ T cells and virus-specific follicular helper T (T_FH_) cells. Overall, these findings pinpoint critical immune effector and regulatory mechanisms essential for a potent, yet harmless resolution of COVID-19 infection.

## Introduction

The global coronavirus disease 2019 (COVID-19) pandemic, caused by the newly emerged severe acute respiratory syndrome coronavirus 2 (SARS-CoV-2) (1), has led to enormous loss of life as well as critical social and economic disruption (2). SARS-CoV-2 infection can lead to diverse clinical fates ranging from severe respiratory failure and shock, leading to death (3–5), to an asymptomatic or mild disease course (6–8). Multiple distinct mechanisms are responsible for the clinical heterogeneity and complex immunopathogenesis of COVID-19 (9). Previous studies have revealed immune signatures of adverse clinical outcomes that are critical for the identification of prognostic factors (9–13) and tractable therapeutic targets (12–14). However, it is crucially important that we identify the mechanisms that promote an effective, yet harmless resolution of infection and confirm the presence of protective immunity against SARS-CoV-2 in those who have recovered from mild COVID-19.

A productive SARS-CoV-2 infection must commence with viral entry into the host cells through the binding of its spike glycoprotein receptor-binding domain (RBD) to the human angiotensin-converting enzyme 2 (hACE-2) receptor (15). Immunologically, the SARS-CoV-2 pseudo-virus neutralization titers that indicate protection match the spike and RBD IgG titers post-COVID-19 infection (16). This protective response is consistent with an observed skewing of the spike-specific CD4^+^ T cells towards a circulating T follicular helper (T_FH_) profile, which is crucial for antibody production (17), in convalescent COVID-19 individuals (18). Unsurprisingly, the spike glycoprotein has been a key target in SARS-CoV-2 vaccine development (19). Its pathologic importance has been further consolidated with the successes of the Pfizer-BioNTech (20) and Moderna (21) COVID-19 mRNA vaccines that encode the full spike glycoprotein sequence. Both vaccines have completed Phase III clinical trials and are approved by the US Food and Drug Administration for emergent use. Hence, understanding the immunological memory response to SARS-CoV-2, in particular spike RBD, will be useful for prognostication and vaccine development.

In this study, we aimed to gain a comprehensive understanding of the convalescent COVID-19 immune architecture. For this purpose, we analyzed the COVID-19 immune response in peripheral blood mononuclear cells (PBMCs) using an adaptation of a standardized mass cytometry protocol based on the Extended Polydimensional Immunome Characterization (EPIC) platform (**Supplemental Tables 1**, **2**) (22). We characterized the immunological profiles of 19 convalescent COVID-19 patients whose infections were confirmed by nasal swab SARS-CoV-2 polymerase chain reaction (PCR) (**Supplemental Table 3**). This cohort was specifically selected for their mild disease course that is likely to be underpinned by an effective immunoregulatory mechanism, an assertion supported by a report of reduced SARS-CoV-2 reactive regulatory T (T_reg_) cell in hospitalized compared with non-hospitalized COVID-19 patients using single-cell transcriptomic analysis (11). Notably, we discovered a robust effector T (T_eff_) cell recall response accompanied by a highly specialized T_reg_ cell subset. Both of these T cell subsets were reactive to a peptide pool covering the RBD of the SARS-CoV-2 spike glycoprotein. This combination of antigenic specificity and dichotomic function implies the existence of an immunological rheostat that provides immunocompetence and prevents excessive inflammatory damage in patients with a benign clinical course.

## Materials and Methods

### Participants and Study Approval

Peripheral blood samples were obtained from COVID-19 patients (n = 19) in the convalescent phase. Written informed consent was obtained from COVID-19 patients who provided clinical data and biological samples (the ‘PROTECT’ study). The study protocol was approved by the ethics committee of the National Healthcare Group (Ref: 2012/00917). Samples from pediatric and adult healthy donors (HD) were collected before September 2019, prior to the COVID-19 pandemic. HD samples were acquired from KK Women’s and Children’s Hospital with ethical approval from the SingHealth Centralized Institutional Review Board and after informed consent was obtained (Ref: 2019/2194 and 2019/2239).

### Cell Isolation

Blood samples were collected in ethylenediaminetetraacetic acid tubes, from which PBMCs were isolated by density centrifugation using Ficoll-Paque PLUS (GE Healthcare, UK) and subsequently cryopreserved in fetal calf serum (FCS, Gibco, USA) with 10% (v/v) dimethyl sulfoxide (DMSO, Sigma–Aldrich, UK).

### Mass Cytometry

Cryopreserved PBMCs were thawed in Roswell Park Memorial Institute 1640 (RPMI) medium supplemented with 10% (v/v) human serum (Corning, USA) and 1× (v/v) penicillin-streptomycin-glutamine (Gibco, USA). Cells were then resuspended in the same medium and rested for 30 min at 37°C. Subsequently, the cells were harvested and divided into 4 experimental conditions (1): non-stimulation (2), stimulation with phorbol 12-myristate 13 acetate (PMA) and ionomycin (3), stimulation with the SARS-CoV-2 peptide pool and (4) stimulation with the peptide solvent control (**Supplemental Table 4**). PMA and ionomycin (both from Sigma-Aldrich, UK) stimulation was done for 5 h. Brefeldin A and monensin (eBioscience) were added to all groups, except the peptide pool stimulation group, during the last 3 h of the incubation for blockade of protein transport.

The cells in all groups, except the PMA/ionomycin stimulation group, were processed using the standardized EPIC staining protocol as described previously (22). In brief, PBMCs were washed once with cell staining buffer (CSB) (phosphate buffered saline [PBS] with 4% FCS, 2 mM EDTA, 0.05% sodium azide) and centrifuged at 524 ×*g* for 6 min at 4°C. The supernatant was decanted and the cells were stained with cisplatin viability stain (PBS with 10 μM cisplatin) (DVS Sciences, USA) for 5 min on ice. PBMCs were then washed and stained with a quadruplet barcode system comprising of CD45 antibodies conjugated with Y-89, Cd-106, Cd-113, or Sn-115 (23) (**Supplemental Table 1**). PBMCs subjected to peptide and solvent control stimulation were also stained with 40 μl phycoerythrin (PE)-conjugated anti-human TCR α/β (BD Pharmingen, USA), and 5 μl fluorescein isothiocyanate (FITC)-conjugated anti-human TCR γ/δ (Invitrogen, USA) with the barcodes in a 100 μl volume. PBMCs in the unstimulated group were also stained with biotinylated SARS-CoV-2 spike protein RBD, His-tagged (Catalog #: SPD-C52H3, Acro Biosystems, DE, USA) at 4 μg/ml (**Supplemental Table 2**). After incubation on ice for 20 min, PBMCs were washed three times before they were combined and pelleted in preparation for surface staining with the antibody panels (Panel A or B; **Supplemental Tables 1**, **2**). PBMCs were first stained with lanthanide-conjugated surface marker antibodies on ice for 25 min in a final reaction volume of 150 μl. After washing twice (initially with CSB and then with 1× PBS), PBMCs were fixed and permeabilized in 1 mL of fixation/permeabilization buffer (eBioscience, USA) for 45 min on ice. PBMCs were then washed twice with permeabilization wash buffer (eBioscience, USA) and centrifuged at 840 ×*g* for 6 min. After decanting the supernatant, PBMCs were stained with lanthanide-conjugated intracellular marker antibodies on ice for 45 min in a final reaction volume of 150 μl. PBMCs were subsequently washed once with permeabilization wash buffer and resuspended in 1× PBS with 1.6% paraformaldehyde (PFA) for 2 days at 4°C prior to data acquisition.

To facilitate future comparative analyses, the staining protocol for PMA/ionomycin stimulation was modified to include an initial 4% paraformaldehyde (PFA) fixation that parallels the viral inactivation step applied to acute SARS-CoV-2 biological samples from patients. The modified protocol first required cisplatin viability staining of PBMCs, followed by pre-staining with lanthanide-conjugated antibodies for selected markers (CD19, CD56, CD8, CD127, CCR7, CXCR3, CD161, CD31; **Supplemental Table 1**) and anti-human TCR γ/δ (Invitrogen, USA) for 15 min, room temperature (RT). After two washes with 1× PBS, PBMCs were fixed in 4% PFA (30 min, RT) and resuspended in CSB before reversion to the standardized EPIC staining protocol with omission of the antibodies used in pre-staining step.

On the day of data acquisition, the cells were pelleted and stained with 500 μL DNA intercalator (DVS Sciences, USA) diluted in 1.6% PFA/1× PBS for 20 min, RT. The cells were then washed once with CSB and once with UltraPure™ DNase/RNase-Free Distilled Water (Invitrogen, USA). The pelleted cells were resuspended to a density of 10^6^/mL in UltraPure DNase/RNase-Free Distilled Water with 10% (v/v) EQTM Four Element Calibration Beads (Fluidigm, USA) in accordance to the manufacturer’s instructions. Data acquisition was then performed using a Helios mass cytometer (Fluidigm, USA).

### Processing of Data Output from Helios Mass Cytometer

The Helios-generated output files were normalized using EQTM Four Element Calibration Beads (24). The live single cell events singlets were gated *via* 2 steps: first by identifying singlets *via* a bivariate plot of DNA intercalator versus event length, and next by detecting singlets that are negative for cisplatin as previously described (23). De-barcoding was carried out using a bivariate gating strategy in FlowJo (Version 10.7.1, Becton, Dickinson & Company, USA) and exported for unsupervised analysis.

### Peptide Design

SARS-CoV-2 epitopes were predicted using the protein sequence derived from the reference SARS-CoV-2 isolate, Wuhan-Hu-1 (NCBI: YP_009724390.1) and tools available on the Immune Epitope Database and Analysis Resource (IEDB) as previously described (25). A pool of seven peptides covering the RBD of the SARS-CoV-2 spike glycoprotein were synthesized to identify T cell subsets critical to protective immunity (**Supplemental Table 4**) (GenScript, USA). These putative T cell epitopes were previously predicted to be immunodominant (25).

### Unsupervised Analysis

#### Batch Normalization

To mitigate the effect of batch variations between mass cytometry runs, we applied batch-wise scaling as a pre-processing step before clustering as described previously (22). Batch-wise scaling involves standard scaling grouped by batches instead of applying to all data. This process achieves centering to zero and unit scaling for the whole dataset as well as for all batch subsets.

#### Clustering

To identify cell populations in an unsupervised manner, flow or mass cytometry analysis using self-organizing maps (FlowSOM) clustering (26) was applied after random down-sampling to 10,000 cell events per subject. FlowSOM is based on self-organizing maps and can be used to process very large datasets. This approach outperforms other methods in terms of speed and accuracy (27). All clustering and dimensionality reduction operations were preceded by hyperbolic arcsine transformation with a scale factor of 5 (asinh5). Batch-wise standard scaling was applied after asinh5 to assign equal weights to all features and reduce technical variation. Protein expression patterns of clusters were examined using dendrogram heat maps constructed using the ‘heatmaply’ R package.

#### Dimensionality Reduction

Non-linear dimensionality reduction was performed using t-Distributed Stochastic Neighbor Embedding (tSNE) (28) to visualize multi-dimensional expression landscapes in two dimensions (2D). ‘Relatedness’ among different clusters was visualised after embedding FlowSOM clustering information onto the 2D tSNE plots.

### SARS-CoV-2 Enzyme-Linked Immunosorbent Assay (ELISA)

SARS-CoV-2 spike protein S1 RBD-specific IgG was quantified using a commercial ELISA kit (Catalog #: MBS398005, MyBioSource Inc., USA) according to the manufacturer’s instructions.

### Statistical Analysis

Verification of FlowSOM clustering frequency (expressed as a percentage of CD45^+^ PBMCs) was performed with bivariate supervised gating in FlowJo (Version 10.7.1, BD, USA). Cell subset frequencies were plotted as the median with interquartile ranges or mean ± standard deviation. Statistical analysis was performed with Mann–Whitney U-tests with no assumption of the underlying data distribution using Prism (Version 7.0e, GraphPad Inc., USA).

## Results

### Restoration of the Convalescent COVID-19 Immunome Toward a Healthy Immunological State

**Figure 1:**
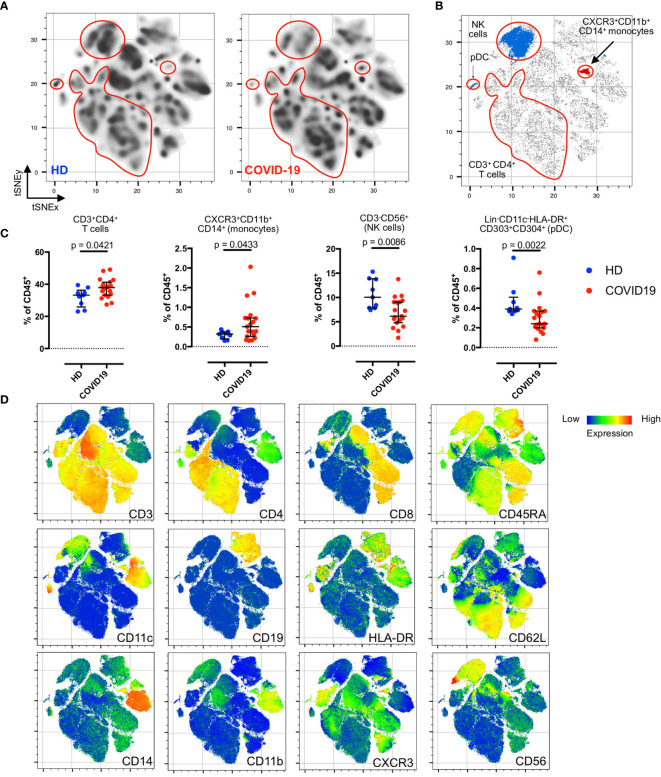
Residual post-COVID-19 immune signatures during the convalescent phase. **(A)** Density plots after t-SNE dimensionality reduction demonstrate a similarity between the convalescent and healthy immunomes. Each density plot is derived from the random sampling of 50,000 single events from the concatenated mass cytometry data from 9 HD and 19 COVID-19 subjects for their respective plot. The red outlines demarcate statistically different cell clusters between COVID-19 patients and HD. **(B)** Total CD3^+^CD4^+^T cells (merged FlowSOM CD3^+^CD4^+^ clusters), CXCR3^+^CD11b^+^CD14^+^ monocytes, CD3^-^CD56^+^ NK cells (merged FlowSOM CD3^-^CD56^+^ clusters) and Lin^-^CD11c^-^HLA-DR^+^CD303^+^CD304^+^ pDC showing statistically significant differences between COVID-19 and HD. Significantly different cell clusters are coloured blue (enriched in HD) or red (enriched in COVID-19 patients). Scatter plot of 280,000 single cell events with 10,000 per subject (n = 19, COVID-19 and n = 9, HD). **(C)** Comparison of FlowSOM-derived cell frequencies of CD3^+^CD4^+^ T cells, CXCR3^+^CD11b^+^CD14^+^ monocytes, CD3^-^CD56^+^ NK cells and pDC between HD and COVID-19 convalescent patients. The FlowSOM-derived cell frequencies strongly correlated with the bivariate gated cell frequencies (**Supplemental Figure 3**), indicating the reliability of the FlowSOM-derived cluster frequencies for statistical inferences. **(D)** t-SNE plots with embedded marker expression included 280,000 single cell events with 10,000 per subject (n = 19, COVID-19 and n = 9, HD). Median and interquartile range (IQR) are shown. Mann–Whitney U (two-tailed) test, *p* < 0.05: statistically significant. Unstimulated PBMCs were stained with COVID-19 panel **(B)** (**Supplemental Table 2**).

We compared the circulatory immune profiles of 19 convalescent COVID-19 patients (4 asymptomatic and 15 with mild disease, median age of 10 with an interquartile range (IQR) of 7 to 15 years, age range of 3 to 36 years and 9 male and 10 female) to healthy donors (HD) of comparable ages (**Supplemental Table 3**). In addition, the main clinical manifestations amongst the symptomatic cases were fever (n = 10), cough/sore throat/rhinorrhea (n = 8), gastrointestinal symptoms (n = 2) and loss of smell/taste (n = 2). All cases were mild and did not require any specific COVID-19 treatment, supplemental oxygen therapy or ventilatory support during their disease course. The biological samples from the HD were collected before September 2019, prior to the COVID-19 pandemic. The convalescent COVID-19 immunome was visualized on a t-SNE (28) plot embedded with cell cluster information identified by FlowSOM (26) clustering (**Figure 1**). This enabled a holistic depiction of the immunome and outlined the “relatedness” of distinct cell clusters in a two-dimensional space.

First, we explored the convalescent COVID-19 immunome without stimulation. As shown in **Figure 1A**, we detected a near-complete restoration of a healthy immunological state, despite several residual changes from the recent infective insult (**Figure 1B**). When compared to unexposed HD, we did not observe any statistically significant differences in the basal populations of unstimulated naïve (CD45RA^+^CD62L^+^), effector (CD45RA^+^CD62L^-^), central memory (T_CM_; CD45RA^-^CD62L^+^) and effector memory (T_EM_; CD45RA^-^CD62L^-^) subsets of the CD3^+^CD4^+^ and CD3^+^CD8^+^ T cells in the COVID-19 patients (**Supplemental Figure 1**). There were no differences between HD and COVID-19 patients in the frequencies of CD19^+^ B cells and their subsets (naïve, transitional, memory, plasmablasts and plasma cells; **Supplemental Figure 2**). However, we noted a concomitant enrichment of CD3^+^CD4^+^ T cells (*p* = 0.0421, two-tailed Mann–Whitney *U*-test) and CXCR3^+^CD11b^+^CD14^+^ monocytes (*p* = 0.0433, two-tailed Mann–Whitney *U*-test), accompanied by a reduction in natural killer (NK) cells (*p* = 0.0086, two-tailed Mann–Whitney *U*-test), and Lin^-^CD11c^-^HLA-DR^+^CD303^+^CD304^+^ plasmacytoid dendritic cells (pDCs; *p* = 0.0022, two-tailed Mann–Whitney *U*-test) in the COVID-19 patients (**Figures 1B–D**). These findings were verified with supervised bivariate gating (**Supplemental Figure 3**).

### Enrichment of IFNγ- and TNFα-Secreting CD4^+^ T_EM_ During COVID-19 Convalescence

**Figure 2:**
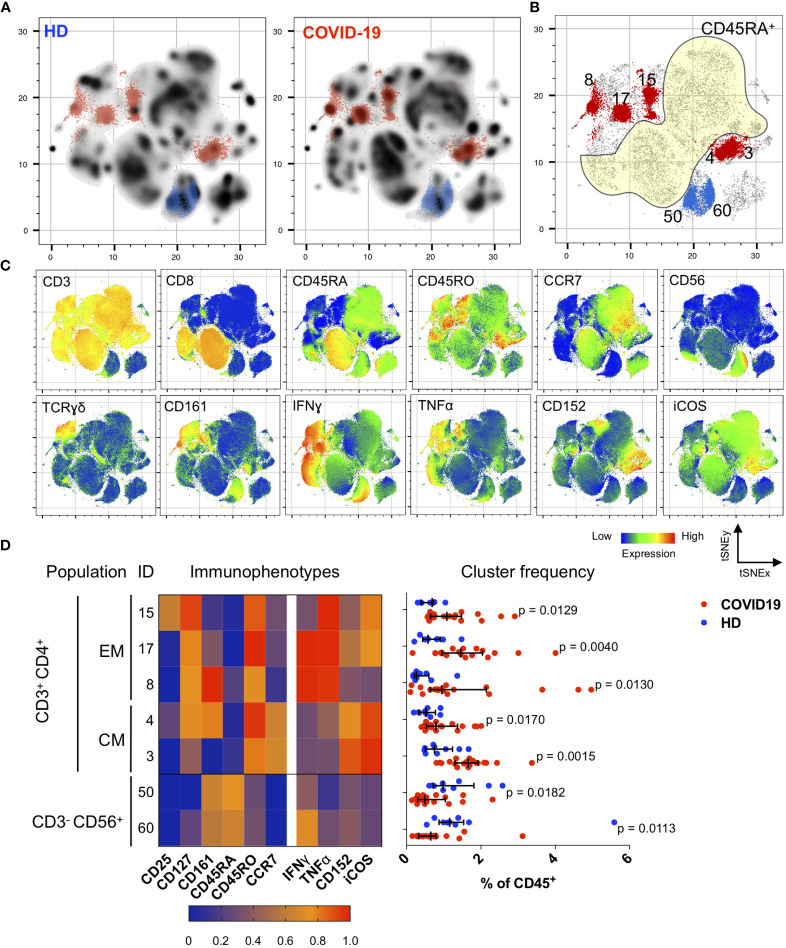
Immunological changes in the memory compartment (CD45RO^+^CD45RA^-^) of the CD3^+^CD4^+^ cell subsets bear mechanistically important functional markers during the convalescent phase. **(A)** Density plots after t-SNE dimensional reduction show a convalescent immunome with visible differences when compared to the healthy immunome. Each density plot is derived from random sampling of 50,000 single events from concatenated mass cytometry data from 9 HD and 1019 COVID-19 subjects for their respective plot. **(B)** Persistent perturbations predominantly involve the CD45RO^+^CD45RA^-^CD3^+^CD4^+^ (outside yellow demarcated area) T cell subset. Clusters showing statistically significant differences are coloured blue (enriched in HD) or red (enriched in disease). These regions are overlaid onto the density plots in **(A)**. Scatter plot of 280,000 single cell events with 10,000 per subject (n = 19, COVID-19 and n = 9, HD). **(C)** t-SNE with embedded marker expression. **(D)** Scaled median (arcsine transformed) marker expression profiles (heat maps) of significant FlowSOM clusters and their frequencies depicted as a percentage of CD45^+^ PBMC (n = 19, COVID-19 and n = 9, HD). Median and IQR are shown. Mann–Whitney U (two-tailed) test, *p* < 0.05 for these seven clusters. Stimulated PBMCs (with PMA and ionomycin) were stained with COVID-19 panel **(A)** (**Supplemental Table 1**).

Given the absence of significant compositional changes in the baseline naïve, effector, T_CM_ and T_EM_ subsets of CD3^+^CD4^+^ and CD3^+^CD8^+^ T cells, we then investigated the extent of functional differentiation within the convalescent COVID-19 immunome by stimulating the samples with PMA and ionomycin (**Figure 2**).

In convalescent COVID-19 patients, we found significant enrichment in the CD45RA^-^ (memory) compartment of the CD4^+^ T cells with stimulation (**Figures 2A, B**). Specifically, we observed an increase in CD4^+^ T_EM_ cells expressing IFNγ and/or TNFα (CD3^+^CD4^+^CD45RA^-^CD45RO^+^CCR7^-^; *p* < 0.05, two-tailed Mann–Whitney *U*-test), and CD4^+^ T_CM_ cells co-expressing CD152 (also known as cytotoxic T lymphocyte-associated antigen 4, CTLA-4) and ICOS (inducible T cell co-stimulator) (CD3^+^CD4^+^CD45RA^-^CD45RO^+^CCR7^+^; *p* < 0.05, two-tailed Mann–Whitney *U*-test) (**Figures 2B–D**). These changes were verified by bivariate gating (**Supplemental Figures 4** and **5**). It is noteworthy that these CD4^+^ T_EM_ cells also expressed CD127, which is the alpha chain of the IL-7 receptor and is known to be involved in memory CD4^+^ T cell survival and proliferation (29, 30) (**Figure 2D** and **Supplemental Figure 4C**). We also observed reductions in two NK cell subsets (as a percentage of CD45^+^ PBMCs): CD3^-^CD56^dim^CD28^hi^IFNγ^-^ (*p* = 0.0061, two-tailed Mann–Whitney *U*-test) and CD3^-^CD56^bright^ (*p* = 0.0491, two-tailed Mann–Whitney *U*-test) (**Supplemental Figure 6**), which might account for the earlier observation that total NK cells were depleted in the convalescent COVID-19 immunome.

### SARS-CoV-2 Specific Immune Rheostat

We then stimulated the samples with a pool of seven peptides covering the RBD of the SARS-CoV-2 spike glycoprotein to identify T cell subsets critical to protective immunity (**Supplemental Table 4**). These putative T cell epitopes were previously predicted to be immunodominant (25). We focused on the SARS-CoV-2 spike glycoprotein RBD since its interaction with the hACE-2 receptor is necessary for viral entry into susceptible cells (15). Furthermore, current evidence supports the immunogenicity of this protein as well as the ability of spike-containing candidate vaccines to elicit a robust CD4^+^ T cell response similar to that induced in response to the natural infection (31).

**Figure 3:**
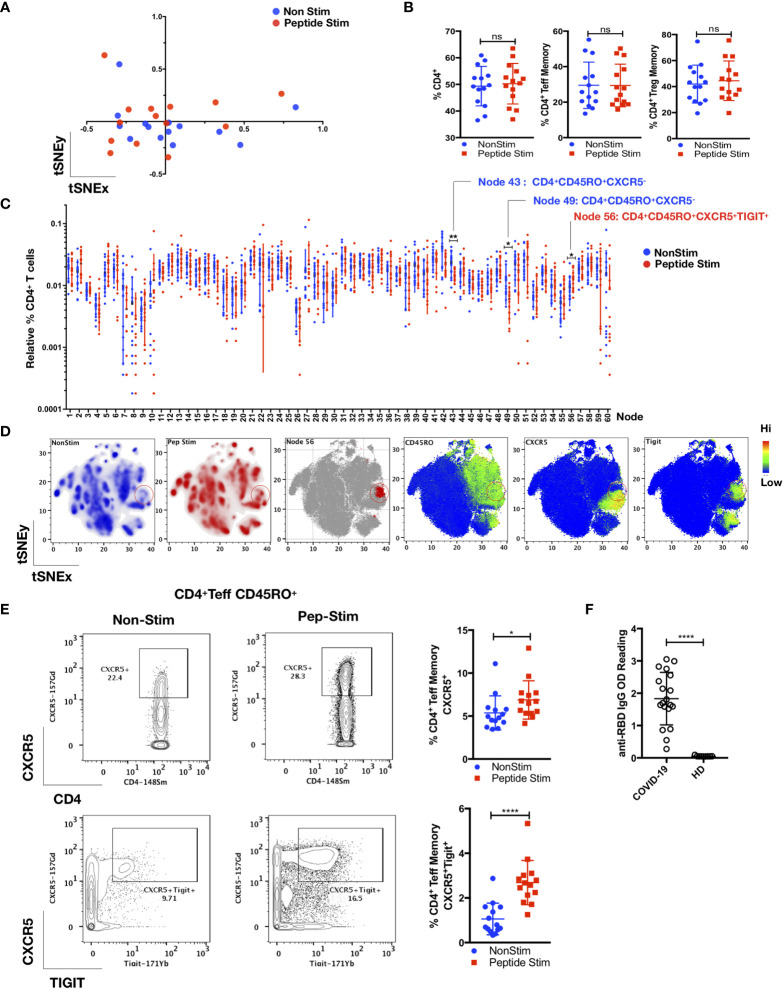
TIGIT^+^ T-follicular helper cells specific for the RBD region of the SARS-CoV-2 spike glycoprotein are present in COVID-19 convalescent patients. PBMCs from convalescent patients (n = 14) were stimulated for 72 h with/without a peptide pool covering the RBD of the SARS-CoV-2 spike glycoprotein and interrogated with COVID panel **(A)**. **(A)** FlowSOM cluster frequencies of CD3^+^CD14^-^CD19^-^CD4^+^CD8^-^ T cells were dimensionally reduced with t-SNE. **(B)** Gated frequencies of CD3^+^CD4^+^, CD3^+^CD4^+^CD25^-/+^FoxP3^-^(T_eff_)CD45RA^-^CD45RO^+^ or CD3^+^CD4^+^CD25^+^FoxP3^+^(T_reg_)CD45RA^-^CD45RO^+^ as percentages of CD3^+^ or CD4^+^ parent lineages. **(C)** Relative percentages of FlowSOM clusters (k = 60) of CD4^+^ T cells, with significant clusters reflected. Data represent the mean ± SD; Mann–Whitney U (two-tailed) test, *p < 0.05, **p < 0.01. **(D)** tSNE maps reflecting density, location and marker expression of cluster 56 (red circles). **(E)** Frequencies of CD4^+^CD25^+/-^FoxP3^-^(T_eff_) CD45RO^+^CD45RA^-^CXCR5^+^ or CD4^+^CD25^+/-^FoxP3^-^(T_eff_)CD45RO^+^CD45RA^-^CXCR5^+^TIGIT^+^ as a percentage of CD4^+^ T cells. Data represent the mean ± SD, Mann–Whitney U (two-tailed) test, *p < 0.05, ****p < 0.0001. **(F)** Anti-RBD IgG OD (optical density) readings of convalescent patient plasma (n = 19) and HD (n = 9). Data represent the mean ± SD, Mann–Whitney U (two-tailed) test, *p < 0.05, ****p < 0.0001. NonStim, peptide solvent control; Peptide Stim, stimulation with peptide pool; ns, statistically not significant.

**Figure 4:**
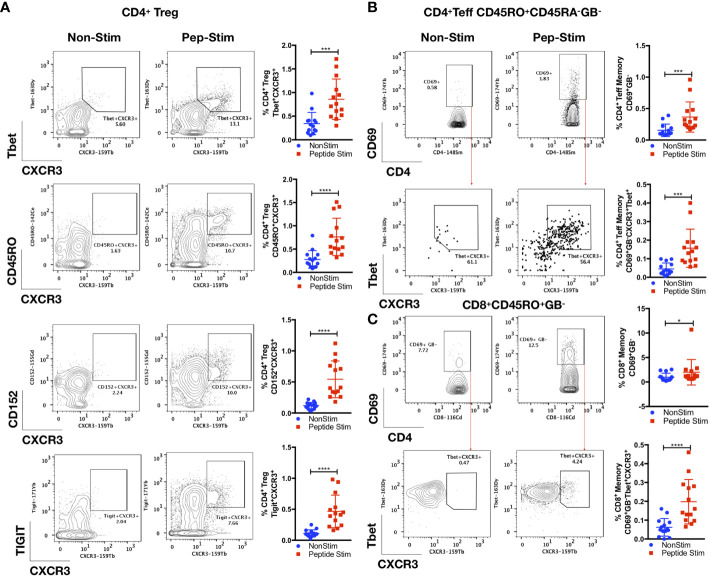
Parallel antigen-specific Th1 responses generated by regulatory and effector subsets. PBMCs from convalescent patients (n = 14) were stimulated for 72 h with/without a peptide pool covering the RBD of the SARS-CoV-2 spike glycoprotein and interrogated with COVID panel **A**. **(A)** Frequencies of CD3^+^CD4^+^CD25^+^FoxP3^+^(T_reg_)CXCR3^+^Tbet^+^, CD3^+^CD4^+^CD25^+^FoxP3^+^(Treg)CXCR3^+^CD45RO^+^, CD3^+^CD4^+^CD25^+^FoxP3^+^(T_reg_)CXCR3^+^CD152^+^ or CD3^+^CD4^+^CD25^+^FoxP3^+^(T_reg_)CXCR3^+^TIGIT^+^ as a percentage of CD4^+^ T cells. **(B)** Frequencies of CD3^+^CD4^+^CD25^+/-^FoxP3^-^(T_eff_)CD45RO^+^CD45RA^-^GB^-^CD69^+^ or CD3^+^CD4^+^CD25^+/-^FoxP3^-^ (T_eff_)CD45RO^+^CD45RA^-^GB^-^CD69^+^Tbet^+^CXCR3^+^. **(C)** CD8^+^CD45RO^+^CD45RA^-^GB^-^CD69^+^ or CD8^+^CD45RO^+^CD45RA^-^GB^-^CD69^+^Tbet^+^CXCR3^+^, as a percentage of CD4^+^ or CD8^+^ parent T cell lineages. Data represent the mean ± SD, Mann–Whitney U (two-tailed) test, *p < 0.05, ***p < 0.001, ****p < 0.0001. NonStim, peptide solvent control; Peptide Stim, stimulation with peptide pool.

As shown in **Figures 3** and **4**, we detected a memory recall response to the peptide pool in the T_FH_, T_reg_ and T_eff_ cell subsets in COVID-19 convalescent patients. The CD4^+^ T cell response is specific, which involves several cell clusters embedded within a stimulated immunological profile that is broadly similar to its control (**Figures 3A–C**). Unsupervised FlowSOM analysis verified by manual bivariate gating revealed enrichment of memory T_FH_ cells (CD4^+^ CD25^+/-^ FoxP3^-^ CD45RO^+^CD45RA^-^CXCR5^+^TIGIT^+^; *p* < 0.05, two-tailed Mann–Whitney *U*-test) (**Figures 3C–E**). Correspondingly, anti-SARS-CoV-2 spike glycoprotein RBD IgG were detected in these COVID-19 convalescent patients (**Figure 3F**).

As shown in **Figure 4**, we also observed an antigen-specific Th1 response generated by distinct T_reg_ and T_eff_ cell subsets in the convalescent COVID-19 patient samples. There was robust enrichment of a Tbet^+^CXCR3^+^ T_reg_ subset (CD3^+^CD19^-^CD14^-^CD4^+^CD25^+^FoxP3^+^Tbet^+^CXCR3^+^; *p* < 0.001, two-tailed Mann–Whitney *U*-test) following peptide stimulation (**Figure 4A** and **Supplemental Figure 7**). This T_reg_ subset displayed a memory phenotype (CD45RO^+^; *p* < 0.0001, two-tailed Mann–Whitney *U*-test) and exhibited a functionally suppressive phenotype (CD152^+^; *p* < 0.0001, two-tailed Mann–Whitney *U*-test) that have been shown to maintain suppression in a pro-inflammatory T_h1_ environment (32) (TIGIT^+^; *p* < 0.0001, two-tailed Mann–Whitney *U*-test) (**Figure 4A**). We observed a parallel increase in the proportion of Tbet^+^CXCR3^+^CD4^+^ (CD3^+^CD19^-^CD14^-^CD4^+^CD25^+/-^FoxP3^-^CD45RO^+^CD69^+^GB^-^Tbet^+^CXCR3^+^) and CD8^+^ (CD3^+^CD19^-^CD14^-^CD4^-^CD8^+^CD45RO^+^GB^-^Tbet^+^CXCR3^+^) T_eff_ cell subsets (*p* < 0.001; **Figure 4B**, and *p* < 0.0001; **Figure 4C** respectively, two-tailed Mann–Whitney *U*-test). No correlations were found between the anti-SARS-CoV-2 spike glycoprotein RBD IgG level with the memory T_FH_ cells (CD4^+^ CD25^+/-^ FoxP3^-^ CD45RO^+^CD45RA^-^CXCR5^+^TIGIT^+^), the Tbet^+^CXCR3^+^ T_reg_ subset (CD3^+^CD19^-^CD14^-^CD4^+^CD25^+^FoxP3^+^Tbet^+^CXCR3^+^), as well as the Tbet^+^CXCR3^+^CD4^+^ (CD3^+^CD19^-^CD14^-^CD4^+^CD25^+/-^FoxP3^-^CD45RO^+^CD69^+^GB^-^Tbet^+^CXCR3^+^) and CD8^+^ (CD3^+^CD19^-^CD14^-^CD4^-^CD8^+^CD45RO^+^GB^-^Tbet^+^CXCR3^+^) T_eff_ cell subsets (**Supplemental Figure 8**).

## Discussion

In this study, we demonstrated that existence of strong memory recall responses consisting of cell types with identical antigen specificity and dichotomic functional roles in convalescent COVID-19 patients. These findings strongly suggest the existence of a competent immune response against COVID-19 that is tightly controlled by regulatory mechanisms. These data provide compelling evidence of fine modulation of the intensity of virus-specific immune responses that facilitate viral clearance without triggering excessive inflammation. Thus, this mechanism represents an antigen-specific immune rheostat that is central to disease resolution.

While there have been other reports of the immunological profiles of convalescent COVID-19 patients (14, 30, 31, 33–35), our study builds upon this fundamental information to provide several new insights. First, our findings indicate the existence of an immune rheostat orchestrated by a Tbet^+^CXCR3^+^ T_reg_ subset (CD3^+^CD19^-^CD14^-^CD4^+^CD25^+^FoxP3^+^Tbet^+^CXCR3^+^) that is known to be functionally stable in a pro-inflammatory environment containing high levels of IFNγ (36), it can be speculated that enrichment of such a subset will balance the actions of its corresponding Tbet^+^CXCR3^+^ CD4^+^ and CD8^+^ T_eff_ subsets shown in this study. This mechanism underlies the regulation of the initial antigenic-specific immune response against COVID-19 and highlights a protective immunotype (composite of immune cell changes) consisting of hyperactive CD4+ and CD8+ T cells, reduced circulating T_FH_, and a robust plasmablast response that is positively correlated with disease severity while another immunotype consisting of minimal T and B cell activation (14). Notably, the association of this immunotype with milder disease suggests the need for a controlled and regulated response for infection clearance and disease resolution. This study builds on current knowledge regarding SARS-CoV-2-specific T cell responses, of which the importance of the T_eff_ and T_reg_ compartments tends to be illustrated in isolation than concomitantly assessed (18, 30, 31, 35, 37, 38). Nevertheless, they are largely consistent with our hypothesis of a functional T_eff_-T_reg_ immune rheostat in mild COVID-19 and recovery, especially when patients with severe COVID-19 had a more robust SARS-CoV-2-specific T_eff_ response and diminished T_reg_ numbers compared to those with mild disease (11, 39). Further studies are warranted to determine how dysfunction in this viral-specific Tbet^+^CXCR3^+^ T_reg_ cell subset can lead to an adverse clinical fate. Additionally, antigen-specific T_reg_ cells can attenuate antiviral immunity in other respiratory infections, such as observed in a murine influenza infection model (40) and dampen the immune response to vaccination (41). As such, the influence of T_reg_ cell subsets should be evaluated in ongoing SARS-CoV-2 vaccine trials.

Second, we identified shared expression of CXCR3 in multiple cell subsets that were enriched during COVID-19 convalescence. This observation indicates that these populations are recruited to the target microenvironment. Previous studies have indicated that CXCR3^+^ monocytes and antigen-specific memory T cell subsets function collectively to promote successful disease resolution (42). Evidence of the role of CXCR3^+^ T_FH_ cells emerged in a prior COVID-19 study in which a positive correlation was identified between the presence of CXCR3^+^ circulating T_FH1_ cells (defined as CD4^+^CD45RA^-^CXCR5^+^CXCR3^+^CCR6^-^) and the circulating titre of SARS-CoV-2-specific antibodies (IgG specific for viral nucleocapsid and spike proteins; n = 13 adults) (34). In other respiratory viral diseases such as influenza, CXCR3^+^ T_FH_ cells were shown to correlate positively with the development of protective antibody responses (43). However, in our study, we found no correlation between the memory T_FH_ (CD4^+^CD45RO^+^CXCR5^+^TIGIT^+^), CD4^+^ Treg Tbet^+^CXCR3^+^, CD4^+^ T_eff_ memory CD69^+^GB^-^Tbet^+^CXCR3^+^ and CD8^+^ T_eff_ memory CD69^+^GB^-^Tbet^+^CXCR3^+^ with the anti-RBD IgG antibody level. This discordance may be attributed to distinct immune response kinetics in the context of a mild COVID-19 infection. The anti-RBD IgG titre enumerates residual antibodies to the original infection. Conversely, these circulating T_eff_ cell subsets that are reactive to peptide stimulation are enriched as a consequence of a rapid recall response at least *in-vitro* (72 hours). This recall response may be representative of the differential resolution of T_eff_ subsets after the original infection. Such incongruity between antibody titre and T_eff_ subsets has also been noted in other SARS-CoV-2 and influenza vaccination studies, which thereby substantiate our observations (44, 45).

Third, we found a significant increase in TIGIT-expressing CXCR5^+^ memory CD4^+^ T cells following stimulation with SARS-CoV-2 virus-specific peptides. TIGIT^+^ T_FH_ cells are known to secrete IL-4 and IL-21. These cytokines have been shown to promote B cell activation and differentiation into plasmablasts and stimulate IgG production *in vitro* (46). Blockade of TIGIT in a B cell-TIGIT^+^ cT_FH_ co-culture resulted in reduced IL-21 production and plasmablast differentiation (46), indicating that TIGIT expression promotes T_FH_ function. Further characterization of these TIGIT^+^ memory T_FH_ cells can further delineate their role in the humoral immunity against SARS-CoV-2. TIGIT has also been identified as a marker of T_reg_ cells that specifically control Th1 responses and its signalling aids in restoring Th1 T_reg_ suppressor function (32). Thus, further investigation of the role of TIGIT^+^ T_reg_ cell subsets in COVID-19 is also warranted.

Fourth, we demonstrated the enrichment of CD4^+^ T_EM_ cells expressing IFNγ and/or TNFα in the absence of changes in the basal populations of unstimulated naïve, effector, T_CM_ and T_EM_ subsets of the CD4^+^ T cells. This complements the known anti-viral properties of IFNγ (47) and the enrichment of these IFNγ-secreting CD4^+^ T_EM_ cells during COVID-19 convalescence strongly suggests their role in mediating effective and long-lasting viral clearance. Notably, depletion of IFNγ secreting CD4^+^ T cells has been reported in hospitalized COVID-19 patients (48, 49) with a trend towards a greater reduction in severe disease (49).

Fifth, there was no statistical difference in the total CD11b^+^CD14^+^ monocytes between convalescent COVID-19 and HD (**Supplemental Figure 1A**), but a specific CXCR3^+^ CD11b^+^CD14^+^ monocyte subset was significantly increased during disease convalescence (**Figures 1A–C**). This is similar to a previously described CXCR3^+^CD14^+^ monocyte subset that trended higher in mild COVID-19 patients relative to both healthy controls and severe COVID-19 subjects (50). This subset was part of an appropriate inflammatory CD14^+^ monocytic response in mild disease, while a dysfunctional CD14^+^ myeloid compartment characterized by low HLA-DR expression was observed in severe COVID-19. As the known CXCR3 ligands, CXCL9, CXCL10, and CXCL11, are predominantly induced by IFNγ (51), the presence of this CXCR3-expressing CD11b^+^CD14^+^ monocyte subset may be mechanistically related to the synchronous increase in the IFNγ-producing CD4^+^ T cell subset in our convalescent COVID-19 cohort (**Figure 2D**). For the contracted pDC population observed in our study, it likely represents the residual sequelae of SARS-CoV-2 infection where the pDC population is known to be decreased during the acute infection (52).

Finally, it is already known that NK cells are depleted during acute COVID-19 (53, 54), and that different NK immunotypes are related to COVID-19 severity (54). The reduction in the total CD3^-^CD56^+^, CD56^dim^CD28^+^IFNγ^-^ and CD56^bright^ NK cell subsets in our study likely represent the residual effects of acute SARS-CoV-2 infection (**Figure 1** and **Supplemental Figure 6D**). It is noteworthy that despite the mild disease in our cohort, the immunological sequelae can still be detectable 1 to 2 months after the acute SARS-CoV-2 infection.

In this study, we have identified a functionally relevant mechanism of immune regulation that may form the foundation of an effective, yet harmless resolution of SARS-CoV-2 infection. Our findings emphasize the potential translational value of dissecting the convalescent COVID-19 immunome. A comprehensive understanding of this mechanism could be useful for predicting clinical outcomes in COVID-19 patients and to inform correlates of cellular immunity for vaccine development.

## Data Availability Statement

The raw data supporting the conclusions of this article will be made available by the authors, without undue reservation.

## Ethics Statement

The studies involving human participants were reviewed and approved by National Healthcare Group (Ref: 2012/00917) SingHealth Centralized Institutional Review Board (Ref: 2019/2194 and 2019/2239). Written informed consent to participate in this study was provided by the participants’ legal guardian/next of kin.

## Author Contributions

SA, JC, and CY conceived and oversaw the study. JC, KT, CY, BY, DL, DA, TA, CYC, NT, JHL, K-QK, XN, and LR oversaw the ethics approval, subject screening and recruitment. JY, JYL, ST, AL, SP, DG, KNY, SH, NS, CJC, and RF performed the experiments. JY, JYL, and ST oversaw and coordinated the mass cytometry staining. PK (statistician) and MW (statistician) created the bio-informatics pipeline for data analysis. JY, JYL, ST, PK, and MW performed the data analysis. FG and KTY provided scientific input on the analysis. DA, AG, and L-FW coordinated studies in the biocontainment facility. SA, JY, JYL, and ST wrote the manuscript with input from all co-authors. All authors contributed to the article and approved the submitted version.

## Funding

This study was conducted with the provision of grant funding from the SingHealth Duke-NUS Academic Medicine COVID-19 Rapid Response Research AM/COV004/2020 (SA) and AM/COV001/2020 (CY). This research was also supported by the National Research Foundation Singapore under its National Medical Research Council (NMRC) Centre Grant Programme (NMRC/CG/M003/2017) (SA) and is administered by the Ministry of Health, Singapore’s NMRC. Other NMRC grant support, NMRC/TA/0059/2017 (JY), NMRC/MOHIAFCAT2/005/2015 (SA), NMRC/TCR/0015-NCC/2016 (SA), NMRC/OFLCG/002/2018 (SA), CIRG19may0052 (SA), NMRC/CSA-SI/0008/2016 (JC), NMRC/STPRG-FY19-001 (L-FW), NMRC/COVID19RF-001 (BY) and NMRC/COVID19RF-003 (L-FW), is gratefully acknowledged.

## Conflict of Interest

The authors declare that the research was conducted in the absence of any commercial or financial relationships that could be construed as a potential conflict of interest.

## References

[1] Zhang YZ Holmes EC. A Genomic Perspective on the Origin and Emergence of SARS-Cov-2. Cell (2020) 181(2):223–7. 10.1016/j.cell.2020.03.035 PMC7194821 32220310

[2] Chakraborty I Maity P. Covid-19 Outbreak: Migration, Effects on Society, Global Environment and Prevention. Sci Total Environ (2020) 728:138882. 10.1016/j.scitotenv.2020.138882 32335410 PMC7175860

[3] Guan WJ Ni ZY Hu Y Liang WH Ou CQ He JX. Clinical Characteristics of Coronavirus Disease 2019 in China. N Engl J Med (2020) 382(18):1708–20. 10.1056/NEJMoa2002032 PMC7092819 32109013

[4] Wölfel R Corman VM Guggemos W Seilmaier M Zange S Müller MA. Virological Assessment of Hospitalized Patients With Covid-2019. Nature (2020) 581(7809):465–9. 10.1038/s41586-020-2196-x 32235945

[5] Wu Z McGoogan JM. Characteristics of and Important Lessons From the Coronavirus Disease 2019 (Covid-19) Outbreak in China: Summary of a Report of 72 314 Cases From the Chinese Center for Disease Control and Prevention. JAMA (2020) 323(13):1239–42. 10.1001/jama.2020.2648 32091533

[6] He X Lau EHY Wu P Deng X Wang J Hao X. Temporal Dynamics in Viral Shedding and Transmissibility of COVID-19. Nat Med (2020) 26(5):672–5. 10.1038/s41591-020-0869-5 32296168

[7] Wei WE Li Z Chiew CJ Yong SE Toh MP Lee VJ. Presymptomatic Transmission of SARS-CoV-2 - Singapore, January 23-March 16, 2020. MMWR Morb Mortal Wkly Rep (2020) 69(14):411–5. 10.15585/mmwr.mm6914e1 PMC7147908 32271722

[8] Yang R Gui X Xiong Y. Comparison of Clinical Characteristics of Patients With Asymptomatic *vs* Symptomatic Coronavirus Disease 2019 in Wuhan, China. JAMA Netw Open (2020) 3(5):e2010182. 10.1001/jamanetworkopen.2020.10182 32459353 PMC7254178

[9] Laing AG Lorenc A Del Molino Del Barrio I Das A Fish M Monin L. A Dynamic Covid-19 Immune Signature Includes Associations With Poor Prognosis. Nat Med (2020) 26(10):1623–35. 10.1038/s41591-020-1038-6 32807934

[10] Song JW Zhang C Fan X Meng FP Xu Z Xia P. Immunological and Inflammatory Profiles in Mild and Severe Cases of COVID-19. Nat Commun (2020) 11(1):3410. 10.1038/s41467-020-17240-2 32641700 PMC7343781

[11] Meckiff BJ Ramírez-Suástegui C Fajardo V Chee SJ Kusnadi A Simon H. Imbalance of Regulatory and Cytotoxic Sars-CoV-2-Reactive Cd4(+) T Cells in COVID-19. Cell (2020) 183(5):1340–53.e16. 10.1016/j.cell.2020.10.001 PMC7534589 33096020

[12] Hadjadj J Yatim N Barnabei L Corneau A Boussier J Smith N. Impaired Type I Interferon Activity and Inflammatory Responses in Severe Covid-19 Patients. Sci (New York NY) (2020) 369(6504):718–24. 10.1126/science.abc6027 PMC7402632 32661059

[13] Arunachalam PS Wimmers F Mok CKP Perera R Scott M Hagan T. Systems Biological Assessment of Immunity to Mild Versus Severe Covid-19 Infection in Humans. Sci (New York NY) (2020) 369(6508):1210–20. 10.1126/science.abc6261 PMC7665312 32788292

[14] Mathew D Giles JR Baxter AE Oldridge DA Greenplate AR Wu JE. Deep Immune Profiling of COVID-19 Patients Reveals Distinct Immunotypes With Therapeutic Implications. Science (New York NY) (2020) 369(6508):eabc8511. 10.1126/science.abc8511 PMC7402624 32669297

[15] Shang J Ye G Shi K Wan Y Luo C Aihara H. Structural Basis of Receptor Recognition by SARS-Cov-2. Nature (2020) 581(7807):221–4. 10.1038/s41586-020-2179-y PMC7328981 32225175

[16] Dan JM Mateus J Kato Y Hastie KM Yu ED Faliti CE. Immunological Memory to SARS-CoV-2 Assessed for Up to 8 Months After Infection. Science (New York NY) (2021) 371(6529):eabf4063. 10.1126/science.abf4063 PMC7919858 33408181

[17] Breitfeld D Ohl L Kremmer E Ellwart J Sallusto F Lipp M. Follicular B Helper T Cells Express Cxc Chemokine Receptor 5, Localize to B Cell Follicles, and Support Immunoglobulin Production. J Exp Med (2000) 192(11):1545–52. 10.1084/jem.192.11.1545 PMC2193094 11104797

[18] Sekine T Perez-Potti A Rivera-Ballesteros O Strålin K Gorin JB Olsson A. Robust T Cell Immunity in Convalescent Individuals With Asymptomatic or Mild Covid-19. Cell (2020) 183(1):158–68.e14. 10.1016/j.cell.2020.08.017 32979941 PMC7427556

[19] Amanat F Krammer F. Sars-Cov-2 Vaccines: Status Report. Immunity (2020) 52(4):583–9. 10.1016/j.immuni.2020.03.007 PMC7136867 32259480

[20] Polack FP Thomas SJ Kitchin N Absalon J Gurtman A Lockhart S. Safety and Efficacy of the BNT162b2 Mrna Covid-19 Vaccine. N Engl J Med (2020) 383(27):2603–15. 10.1056/NEJMoa2034577 PMC7745181 33301246

[21] Baden LR El Sahly HM Essink B Kotloff K Frey S Novak R. Efficacy and Safety of the Mrna-1273 SARS-Cov-2 Vaccine. N Engl J Med (2021) 384(5):403–16. 10.1056/NEJMoa2035389 PMC7787219 33378609

[22] Yeo JG Wasser M Kumar P Pan L Poh SL Ally F. The Extended Polydimensional Immunome Characterization (Epic) Web-Based Reference and Discovery Tool for Cytometry Data. Nat Biotechnol (2020) 38(6):679–84. 10.1038/s41587-020-0532-1 32440006

[23] Lai L Ong R Li J Albani S. A CD45-based Barcoding Approach to Multiplex Mass-Cytometry (CyTOF). Cytometry Part A: J Int Soc Anal Cytol (2015) 87(4):369–74. 10.1002/cyto.a.22640 PMC4670699 25645694

[24] Finck R Simonds EF Jager A Krishnaswamy S Sachs K Fantl W. Normalization of Mass Cytometry Data With Bead Standards. Cytometry Part A: J Int Soc Anal Cytol (2013) 83(5):483–94. 10.1002/cyto.a.22271 PMC3688049 23512433

[25] Grifoni A Sidney J Zhang Y Scheuermann RH Peters B Sette A. A Sequence Homology and Bioinformatic Approach Can Predict Candidate Targets for Immune Responses to SARS-Cov-2. Cell Host Microbe (2020) 27(4):671–80. 10.1016/j.chom.2020.03.002 PMC7142693 32183941

[26] Van Gassen S Callebaut B Van Helden MJ Lambrecht BN Demeester P Dhaene T. Flowsom: Using Self-Organizing Maps for Visualization and Interpretation of Cytometry Data. Cytometry Part A: J Int Soc Anal Cytol (2015) 87(7):636–45. 10.1002/cyto.a.22625 25573116

[27] Weber LM Robinson MD. Comparison of Clustering Methods for High-Dimensional Single-Cell Flow and Mass Cytometry Data. Cytometry Part A: J Int Soc Anal Cytol (2016) 89(12):1084–96. 10.1002/cyto.a.23030 27992111

[28] van der Maaten L Hinton G. Visualizing Data Using T-Sne. J Mach Learn Res (2008) 9:2579–605.

[29] Kondrack RM Harbertson J Tan JT McBreen ME Surh CD Bradley LM. Interleukin 7 Regulates the Survival and Generation of Memory Cd4 Cells. J Exp Med (2003) 198(12):1797–806. 10.1084/jem.20030735 PMC2194153 14662907

[30] Neidleman J Luo X Frouard J Xie G Gill G Stein ES. Sars-Cov-2-Specific T Cells Exhibit Unique Features Characterized by Robust Helper Function, Lack of Terminal Differentiation, and High Proliferative Potential. bioRxiv: Preprint Server Biol (2020). 10.1101/2020.06.08.138826 PMC7437502 32839763

[31] Grifoni A Weiskopf D Ramirez SI Mateus J Dan JM Moderbacher CR. Targets of T Cell Responses to SARS-CoV-2 Coronavirus in Humans With COVID-19 Disease and Unexposed Individuals. Cell (2020) 181(7):1489–501. 10.1016/j.cell.2020.05.015 PMC7237901 32473127

[32] Lucca LE Axisa PP Singer ER Nolan NM Dominguez-Villar M Hafler DA. Tigit Signaling Restores Suppressor Function of Th1 Tregs. JCI Insight (2019) 4(3):e124427. 10.1172/jci.insight.124427 PMC6413794 30728325

[33] Thevarajan I Nguyen THO Koutsakos M Druce J Caly L van de Sandt CE. Breadth of Concomitant Immune Responses Prior to Patient Recovery: A Case Report of non-Severe Covid-19. Nat Med (2020) 26(4):453–5. 10.1038/s41591-020-0819-2 PMC7095036 32284614

[34] Gong F Dai Y Zheng T Cheng L Zhao D Wang H. Peripheral CD4+ T Cell Subsets and Antibody Response in COVID-19 Convalescent Individuals. J Clin Invest (2020) 130(12):6588–99. 10.1172/jci141054 PMC7685722 32841212

[35] Le Bert N Tan AT Kunasegaran K Tham CYL Hafezi M Chia A. Sars-Cov-2-Specific T Cell Immunity in Cases of COVID-19 and SARS, and Uninfected Controls. Nature (2020) 584(7821):457–62. 10.1038/s41586-020-2550-z 32668444

[36] Levine AG Mendoza A Hemmers S Moltedo B Niec RE Schizas M. Stability and Function of Regulatory T Cells Expressing the Transcription Factor T-Bet. Nature (2017) 546(7658):421–5. 10.1038/nature22360 PMC5482236 28607488

[37] Rydyznski Moderbacher C Ramirez SI Dan JM Grifoni A Hastie KM Weiskopf D. Antigen-Specific Adaptive Immunity to SARS-CoV-2 in Acute Covid-19 and Associations With Age and Disease Severity. Cell (2020) 183(4):996–1012.e19. 10.1016/j.cell.2020.09.038 PMC7494270 33010815

[38] Sattler A Angermair S Stockmann H Heim KM Khadzhynov D Treskatsch S. Sars-Cov-2 Specific T-Cell Responses and Correlations With COVID-19 Patient Predisposition. J Clin Invest (2020) 130(12):6477–89. 10.1172/jci140965 PMC7685725 32833687

[39] Peng Y Mentzer AJ Liu G Yao X Yin Z Dong D. Broad and Strong Memory CD4(+) and CD8(+) T Cells Induced by SARS-CoV-2 in UK Convalescent Individuals Following Covid-19. Nat Immunol (2020) 21(11):1336–45. 10.1038/s41590-020-0782-6 PMC7611020 32887977

[40] Lin PH Wong WI Wang YL Hsieh MP Lu CW Liang CY. Vaccine-Induced Antigen-Specific Regulatory T Cells Attenuate the Antiviral Immunity Against Acute Influenza Virus Infection. Mucosal Immunol (2018) 11(4):1239–53. 10.1038/s41385-018-0004-9 29467445

[41] Brezar V Godot V Cheng L Su L Lévy Y Seddiki N. T-Regulatory Cells and Vaccination “Pay Attention and Do Not Neglect Them”: Lessons From HIV and Cancer Vaccine Trials. Vaccines (2016) 4(3):30. 10.3390/vaccines4030030 PMC5041024 27608046

[42] Maurice NJ McElrath MJ Andersen-Nissen E Frahm N Prlic M. Cxcr3 Enables Recruitment and Site-Specific Bystander Activation of Memory Cd8(+) T Cells. Nat Commun (2019) 10(1):4987. 10.1038/s41467-019-12980-2 31676770 PMC6825240

[43] Bentebibel SE Khurana S Schmitt N Kurup P Mueller C Obermoser G. Icos(+)Pd-1(+)Cxcr3(+) T Follicular Helper Cells Contribute to the Generation of High-Avidity Antibodies Following Influenza Vaccination. Sci Rep (2016) 6:26494. 10.1038/srep26494 27231124 PMC4882544

[44] Co MD Orphin L Cruz J Pazoles P Rothman AL Ennis FA. Discordance Between Antibody and T Cell Responses in Recipients of Trivalent Inactivated Influenza Vaccine. Vaccine (2008) 26(16):1990–8. 10.1016/j.vaccine.2008.02.024 PMC2440689 18339461

[45] Reynolds CJ Swadling L Gibbons JM Pade C Jensen MP Diniz MO. Discordant Neutralizing Antibody and T Cell Responses in Asymptomatic and Mild Sars-CoV-2 Infection. Sci Immunol (2020) 5(54):eabf3698. 10.1126/sciimmunol.abf3698 33361161 PMC8101131

[46] Godefroy E Zhong H Pham P Friedman D Yazdanbakhsh K. Tigit-Positive Circulating Follicular Helper T Cells Display Robust B-cell Help Functions: Potential Role in Sickle Cell Alloimmunization. Haematologica (2015) 100(11):1415–25. 10.3324/haematol.2015.132738 PMC4825309 26250578

[47] Zhou F. Molecular Mechanisms of IFN-gamma to Up-Regulate Mhc Class I Antigen Processing and Presentation. Int Rev Immunol (2009) 28(3-4):239–60. 10.1080/08830180902978120 19811323

[48] Mazzoni A Salvati L Maggi L Capone M Vanni A Spinicci M. Impaired Immune Cell Cytotoxicity in Severe Covid-19 is IL-6 Dependent. J Clin Invest (2020) 130(9):4694–703. 10.1172/jci138554 PMC7456250 32463803

[49] Chen G Wu D Guo W Cao Y Huang D Wang H. Clinical and Immunological Features of Severe and Moderate Coronavirus Disease 2019. J Clin Invest (2020) 130(5):2620–9. 10.1172/jci137244 PMC7190990 32217835

[50] Schulte-Schrepping J Reusch N Paclik D Baßler K Schlickeiser S Zhang B. Severe COVID-19 is Marked by a Dysregulated Myeloid Cell Compartment. Cell (2020) 182(6):1419–40.e23. 10.1016/j.cell.2020.08.001 32810438 PMC7405822

[51] Metzemaekers M Vanheule V Janssens R Struyf S Proost P. Overview of the Mechanisms That May Contribute to the Non-Redundant Activities of Interferon-Inducible Cxc Chemokine Receptor 3 Ligands. Front Immunol (2017) 8:1970. 10.3389/fimmu.2017.01970 29379506 PMC5775283

[52] Peruzzi B Bencini S Capone M Mazzoni A Maggi L Salvati L. Quantitative and Qualitative Alterations of Circulating Myeloid Cells and Plasmacytoid DC in SARS-CoV-2 Infection. Immunology (2020) 161(4):345–53. 10.1111/imm.13254 PMC7692244 32870529

[53] Jewett A. The Potential Effect of Novel Coronavirus SARS-CoV-2 on NK Cells; A Perspective on Potential Therapeutic Interventions. Front Immunol (2020) 11:1692. 10.3389/fimmu.2020.01692 32754162 PMC7365845

[54] Maucourant C Filipovic I Ponzetta A Aleman S Cornillet M Hertwig L. Natural Killer Cell Immunotypes Related to COVID-19 Disease Severity. Sci Immunol (2020) 5(50):eabc6832. 10.1126/sciimmunol.abd6832 PMC7665314 32826343

