# Supplementary material for: A Virus-Specific Immune Rheostat in the Immunome of Patients Recovering From Mild COVID-19
Source: Front Immunol. 2021 May 25;12:674279. doi: 10.3389/fimmu.2021.674279 (PMC8185226; doi:10.3389/fimmu.2021.674279)
Supplement: Supplementary file 1 [file DataSheet_1.pdf]

# *Supplementary Material*

**Supplemental Figure 1.** Increase in total $CD3^{+}CD4^{+}$ T cells with no increase in $CD3^{+}CD8^{+}$ T cells in convalescent COVID-19 patients.

**Supplemental Figure 2.** No changes in the total $CD19^{+}$ B cells and its major subsets.

**Supplemental Figure 3.** Validation of FlowSOM clusters with bivariate supervised gating.

**Supplemental Figure 4.** Validation of $CD3^{+}CD4^{+}$ effector memory (EM) FlowSOM clusters with bivariate supervised gating.

**Supplemental Figure 5.** Validation of $CD3^{+}CD4^{+}$ central memory (CM) FlowSOM clusters with bivariate supervised gating.

**Supplemental Figure 6.** Diversity of $CD3^{-}CD56^{+}$ NK cell subsets captured with COVID-19 panel A (stimulated with PMA/ionomycin).

**Supplemental Figure 7.** Gating strategy of relevant $CD4^{+}$ and $CD8^{+}$ T cell population from a representative COVID-19 convalescent patient stimulated with peptide pool for 72 hours.

**Supplemental Figure 8.** Correlation of $CD4^{+}CD25^{+/-}FoxP3^{-}(T_{eff})CD45RO^{+}CD45RA^{-}CXCR5^{+}TIGIT^{+}$, $CD3^{+}CD4^{+}CD25^{+}FoxP3^{+}(T_{reg})CXCR3^{+}Tbet^{+}$, $CD3^{+}CD4^{+}CD25^{+/-}FoxP3^{-}(T_{eff})CD45RO^{+}CD45RA^{-}GB^{-}CD69^{+}Tbet^{+}CXCR3^{+}$ and $CD8^{+}CD45RO^{+}CD45RA^{-}GB^{-}CD69^{+}Tbet^{+}CXCR3^{+}$ cell subsets with anti-RBD IgG OD (optical density) readings.

**Supplemental Table 1.** Antibodies used for mass cytometry (COVID-19 Panel A).

**Supplemental Table 2.** Antibodies used for mass cytometry (COVID-19 Panel B).

**Supplemental Table 3.** Clinical characteristics of COVID-19 patients.

**Supplemental Table 4.** Predicted T cell epitopes from SARS-CoV-2 spike glycoprotein.

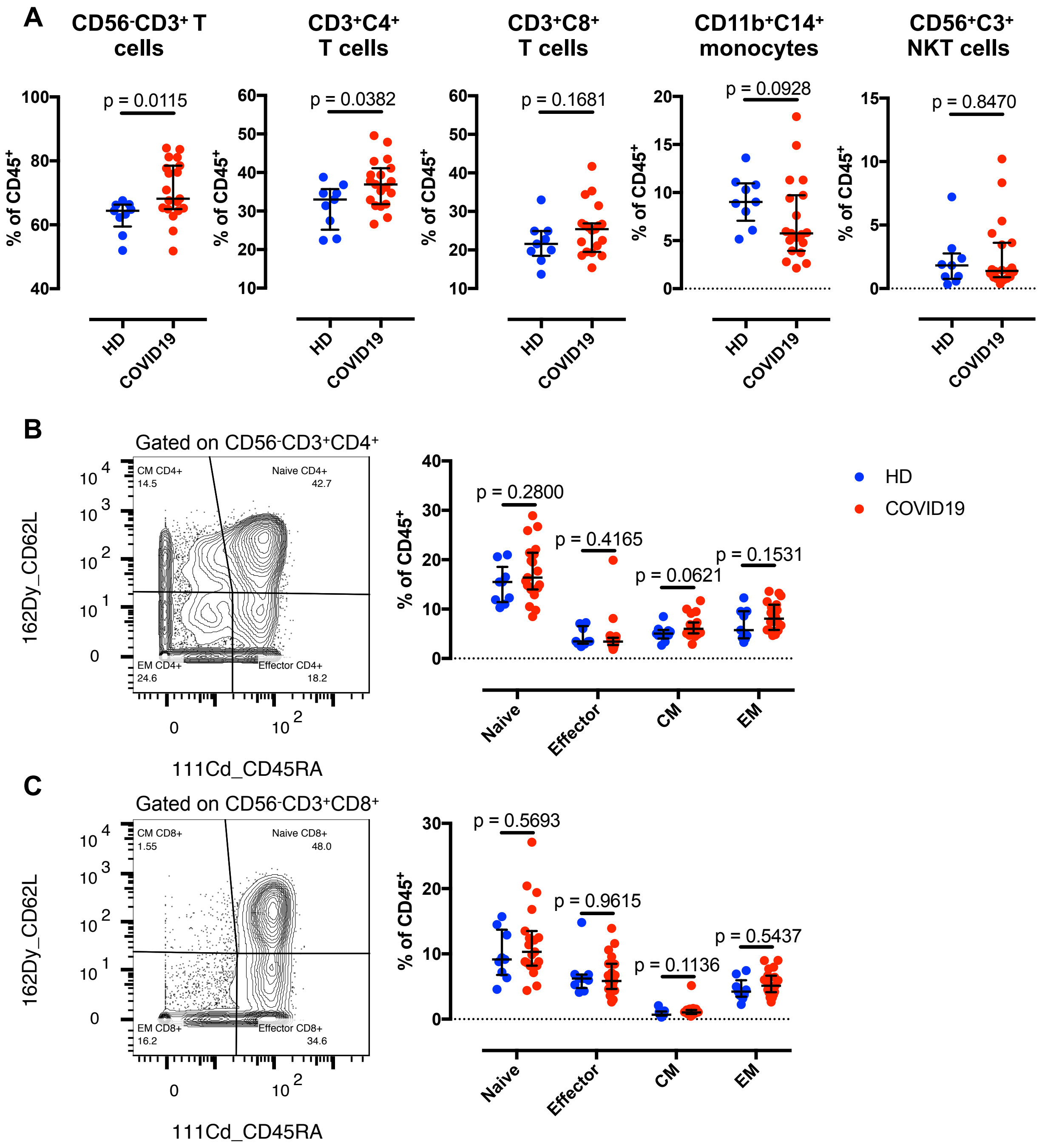


**Supplemental Figure 1.** Increase in total CD3$^+$CD4$^+$ T cells with no increase in CD3$^+$CD8$^+$ T cells in convalescent COVID-19 patients. **(A)** The statistically significant increase in CD56$^-$CD3$^+$CD4$^+$ T cell results in an increase in total CD56$^-$CD3$^+$ T cell subset during the convalescent phase. No significant increases in CD3$^+$CD8$^+$ T cells, CD11b$^+$CD14$^+$ monocytes and CD3$^+$CD56$^+$ NKT cells were detected. **(B)** Representative gates placement for the CD56$^-$CD3$^+$CD4$^+$ population to determine frequencies of naive, effector, CM and EM CD4$^+$ T cells. Although the frequency of the CD56$^-$CD3$^+$CD4$^+$ T cell subset was statistically increased in COVID-19, there were no significant difference in the major CD4$^+$ T cell subsets. **(C)** Bivariate gate placement to determine frequencies of naive, effector, CM and EM CD8$^+$ T cells. Unstimulated PBMC interrogated with COVID panel B. Median and IQR are shown. Mann-Whitney U (two-tailed) test, $p < 0.05$: statistically significant.

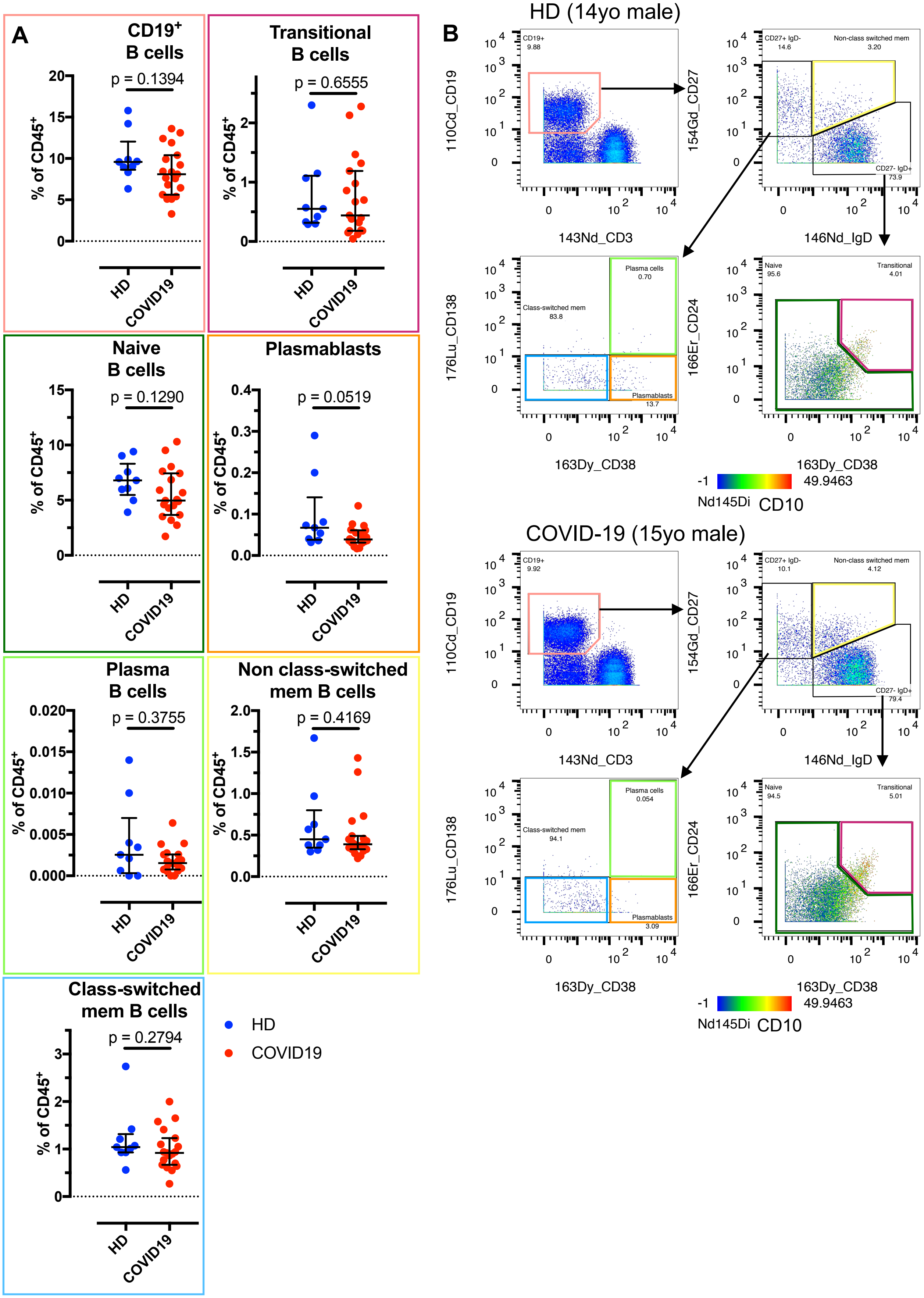


**Supplemental Figure 2.** No changes in the total $CD19^+$ B cells and its major subsets. **(A)** No statistically significant differences were observed in the $CD19^+$ B cell population and its cell subsets. **(B)** Bivariate gate placement to determine frequencies of the B cell subsets in a diseased subject and HD. Unstimulated PBMCs were interrogated with COVID panel B. Similar coloured gates in the bivariate plots and the boxes denote the same population. Median and IQR are shown. Mann-Whitney U (tow-tailed) test, $p < 0.05$: statistically significant.

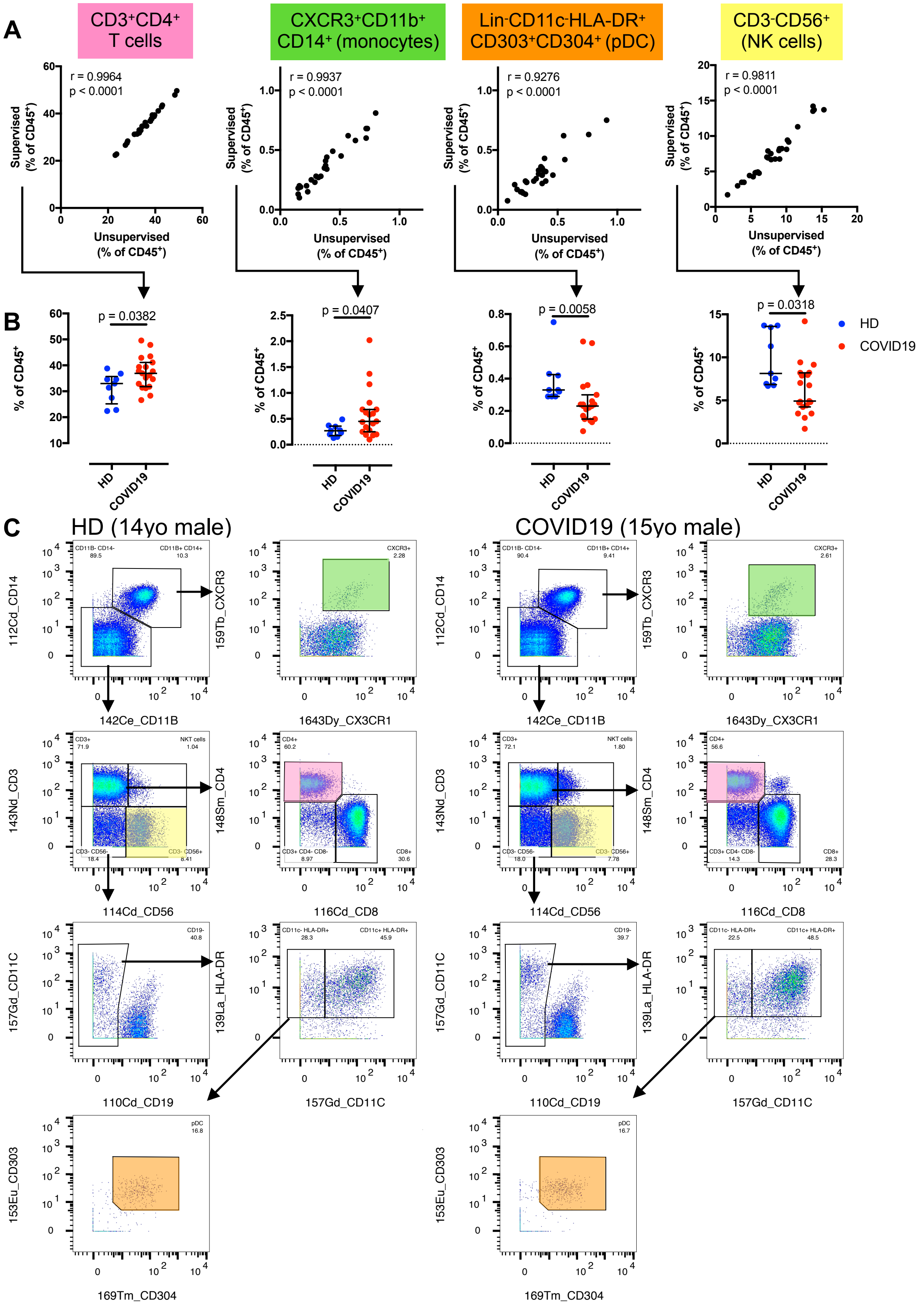


**Supplemental Figure 3.** Validation of FlowSOM clusters with bivariate supervised gating. **(A)** Strong correlation exists between the unsupervised FlowSOM cluster frequencies and supervised bivariate cell frequencies for CXCR3$^+$CD11b$^+$CD14$^+$ monocytes, Lin$^-$CD11c$^-$HLA-DR$^+$CD303$^+$CD304$^+$ pDC, CD3$^-$CD56$^+$ NK cells and CD3$^+$CD4$^+$ T cells. **(B)** Cell frequencies from bivariate gating demonstrate similar results as the FlowSOM-derived cluster frequencies. **(C)** Representative gating strategy for these 4 subsets from a HD and a COVID-19 patient in the convalescent phase. The same cell population is denoted by identical coloured gate in the bivariate plot and title box. R: Pearson's correlation coefficient. Median and IQR are shown. Mann-Whitney U (two-tailed) test, $p < 0.05$: statistically significant.

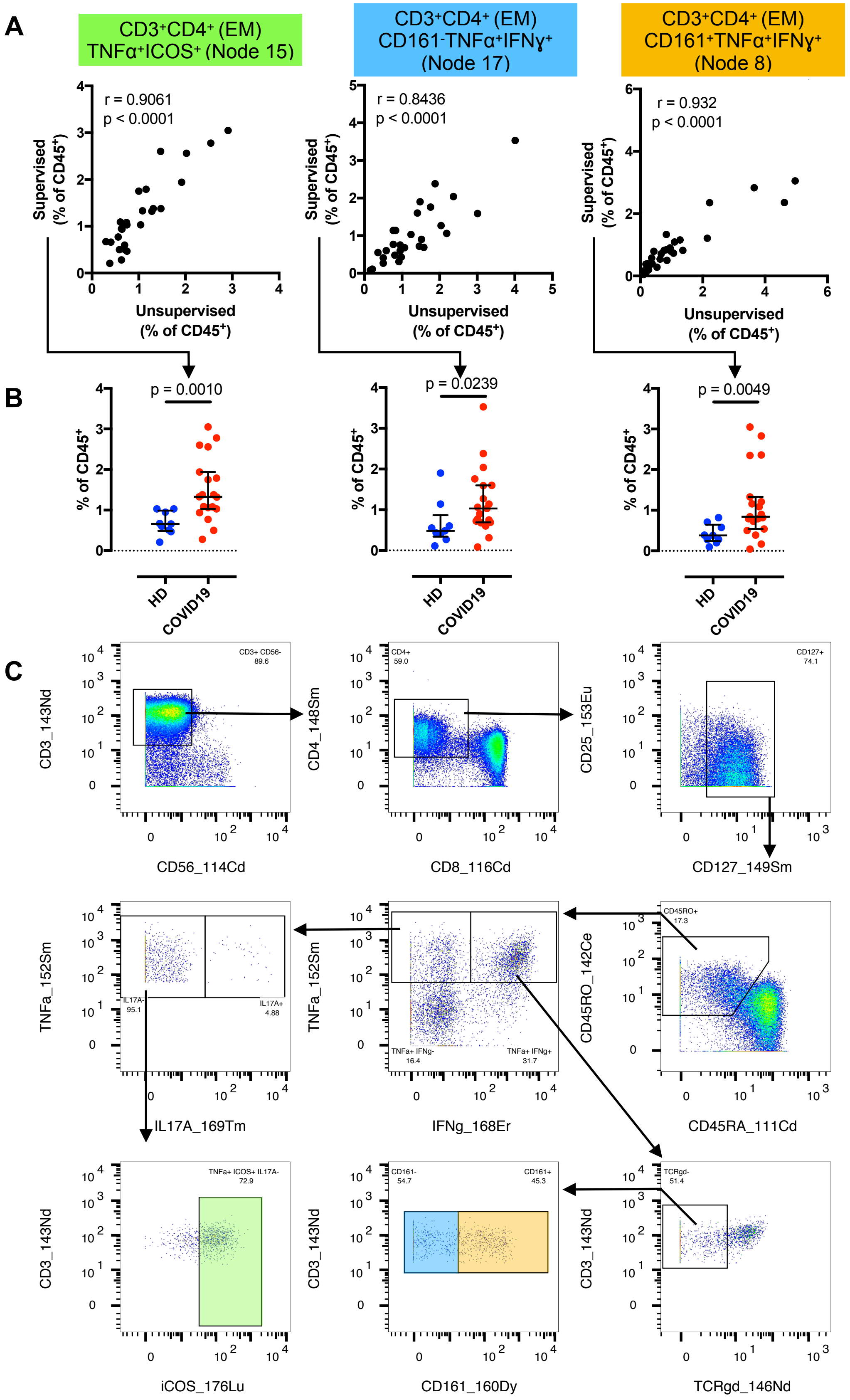


**Supplemental Figure 4.** Validation of CD3+CD4+ effector memory (EM) FlowSOM clusters with bivariate supervised gating. **(A)** Strong correlation exists between unsupervised FlowSOM cluster frequencies and supervised bivariate cell frequencies for the 3 FlowSOM clusters (nodes 15, 17 and 8). **(B)** Cell frequencies from bivariate gating demonstrate similar results to data from unsupervised FlowSOM derived cluster frequencies (Figure 2D). **(C)** Representative gating strategy for these 3 subsets from a COVID-19 patient in the convalescent phase. The same cell population is denoted by identical coloured gate in the bivariate plot and title box. R: Pearson's correlation coefficient. Median and IQR are shown. Mann-Whitney U (two-tailed) test, $p < 0.05$: statistically significant.

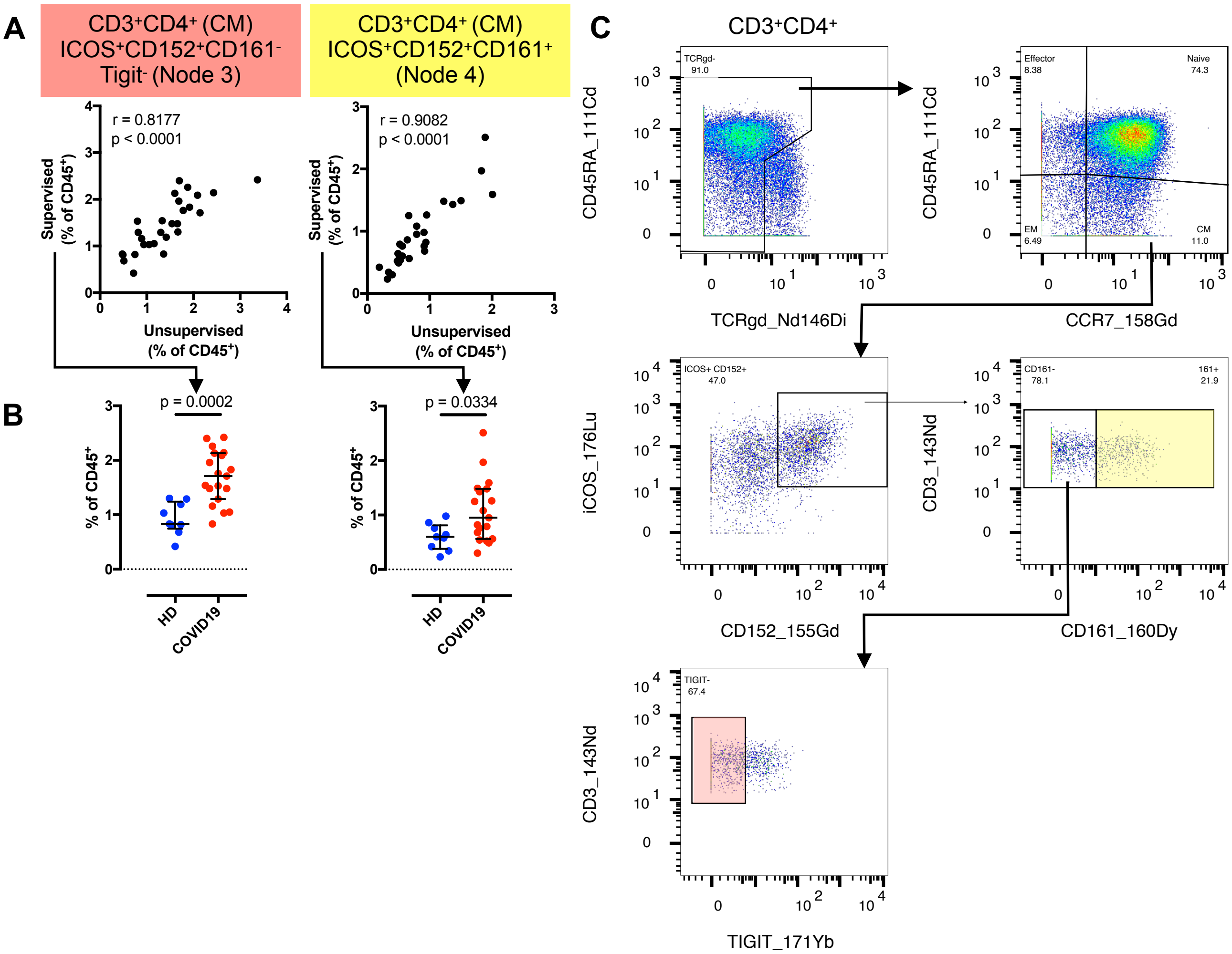


**Supplemental Figure 5.** Validation of CD3+CD4+ central memory (CM) FlowSOM clusters with bivariate supervised gating. **(A)** Strong correlation exists between the unsupervised FlowSOM cluster frequencies and supervised bivariate cell frequencies for the 2 FlowSOM clusters (nodes 3 and 4). **(B)** Cell frequencies from bivariate gating demonstrating similar results as data from unsupervised FlowSOM-derived cluster frequencies. **(C)** Representative gating strategy for these 2 subsets from a COVID-19 patient in the convalescent phase. R: Pearson's correlation coefficient. The same cell population is denoted by identical coloured gate in the bivariate plot and title box. Median and IQR are shown. Mann-Whitney U (two-tailed) test, $p < 0.05$: statistically significant.

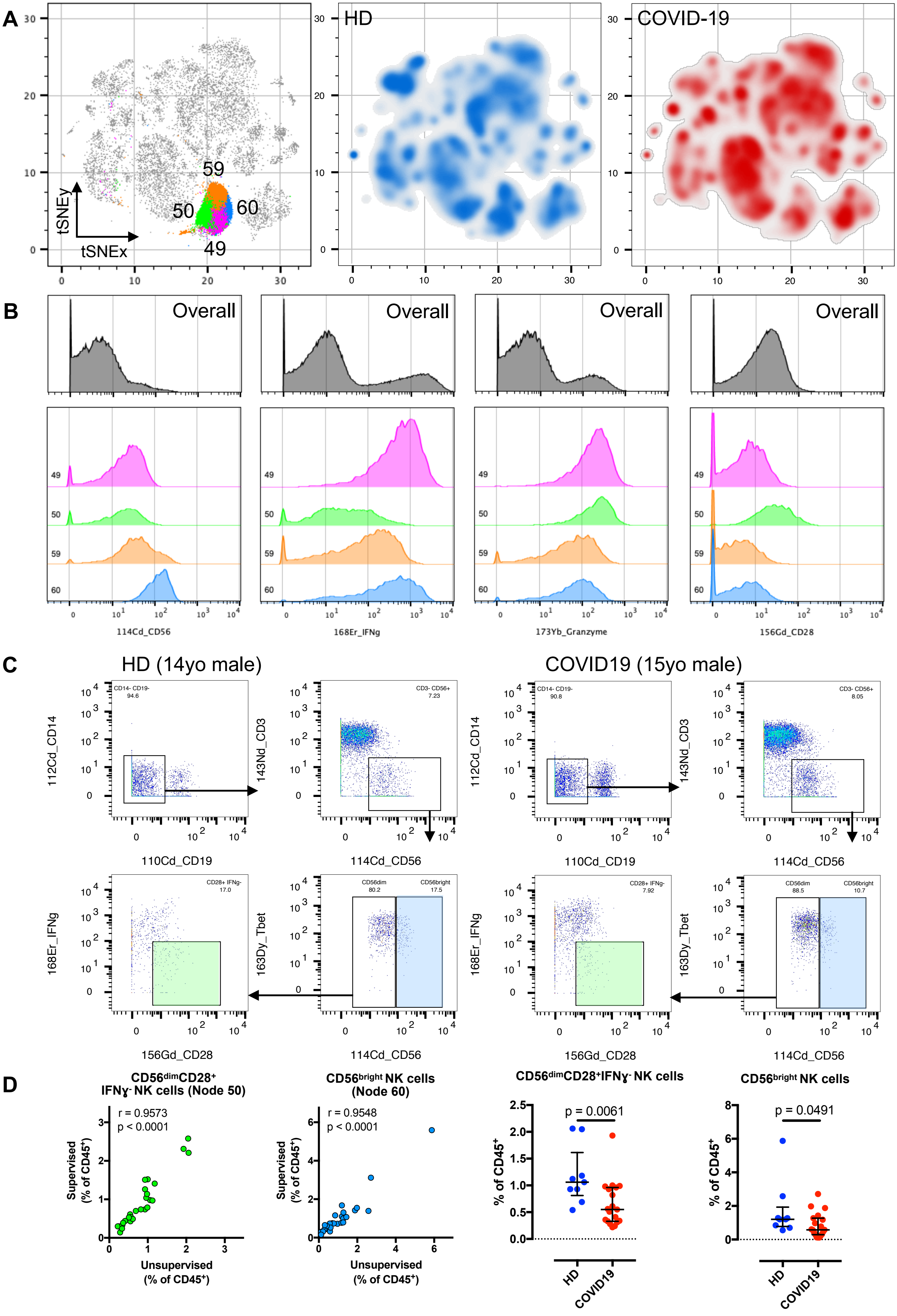
A
HD
COVID-19
tSNEx
tSNEy
59
50
60
49
B
Overall
114Cd_CD56
168Er_IFNg
173Yb_Granzyme
156Gd_CD28
C
HD (14yo male)
COVID19 (15yo male)
112Cd_CD14
110Cd_CD19
143Nd_CD3
114Cd_CD56
168Er_IFNg
156Gd_CD28
163Dy_Tbet
CD14- CD19-
CD3- CD56+
CD28+ IFNg-
CD56dim
CD56bright
D
CD56dimCD28+ IFNɣ- NK cells (Node 50)
r = 0.9573
p < 0.0001
CD56bright NK cells (Node 60)
r = 0.9548
p < 0.0001
Supervised (% of CD45+)
Unsupervised (% of CD45+)
CD56dimCD28+IFNɣ- NK cells
p = 0.0061
CD56bright NK cells
p = 0.0491
% of CD45+
HD
COVID19

**Supplemental Figure 6.** Diversity of CD3$^{-}$CD56$^{+}$ NK cell subsets captured with COVID-19 panel A (stimulated with PMA/ionomycin). **(A)** Density plots after t-SNE dimensional reduction with 4 distinct NK cell FlowSOM clusters being highlighted (cluster/node number 49, 50, 59 and 60). Density plots depicting the immunome of HD (blue) and COVID-19 patients (red) plotted with 50,000 single cell events each. **(B)** Expression histograms of CD28, CD56, granzyme B and IFNɣ of all cells (overall) compared to FlowSOM cluster numbers 49, 50, 59 and 60. **(C)** Representative gating strategy from a HD and a COVID-19 patient in the convalescent phase for the 2 clusters (50 and 60) that were statistically reduced in COVID-19. **(D)** Node 50 denotes the CD3$^{-}$CD56$^{dim}$CD28$^{hi}$IFNɣ$^{-}$ and node 60 denotes the CD3$^{-}$CD56$^{bright}$ NK cells. The FlowSOM derived cell frequencies strongly correlated with the bivariate gated cell frequencies and are different between the HD and COVID-19. Median and IQR are shown. Mann-Whitney U (two-tailed) test, $p < 0.05$: statistically significant.

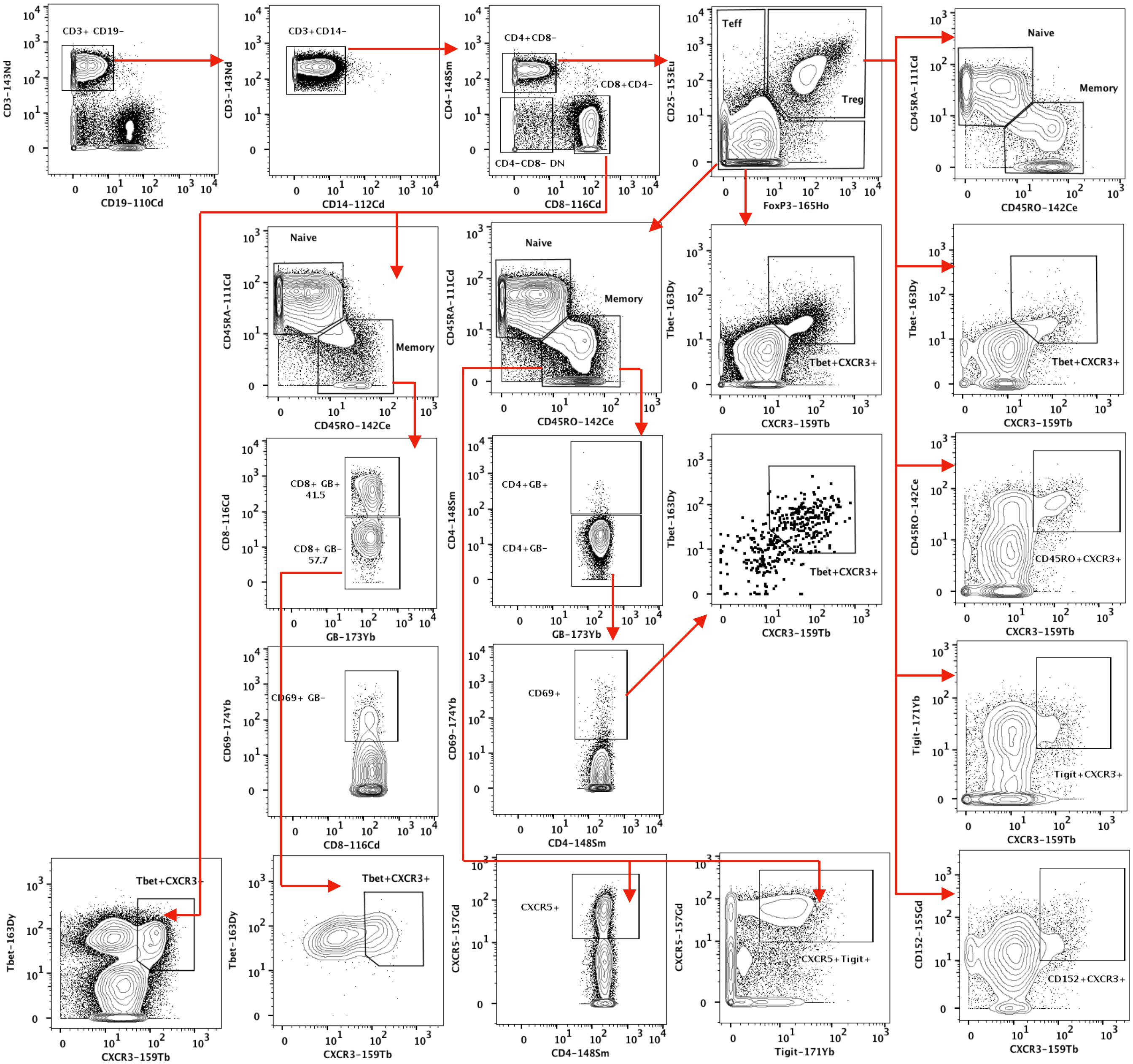


**Supplemental Figure 7.** Gating strategy of relevant $CD4^+$ and $CD8^+$ T cell population from a representative COVID-19 convalescent patient stimulated with peptide pool for 72 hours.

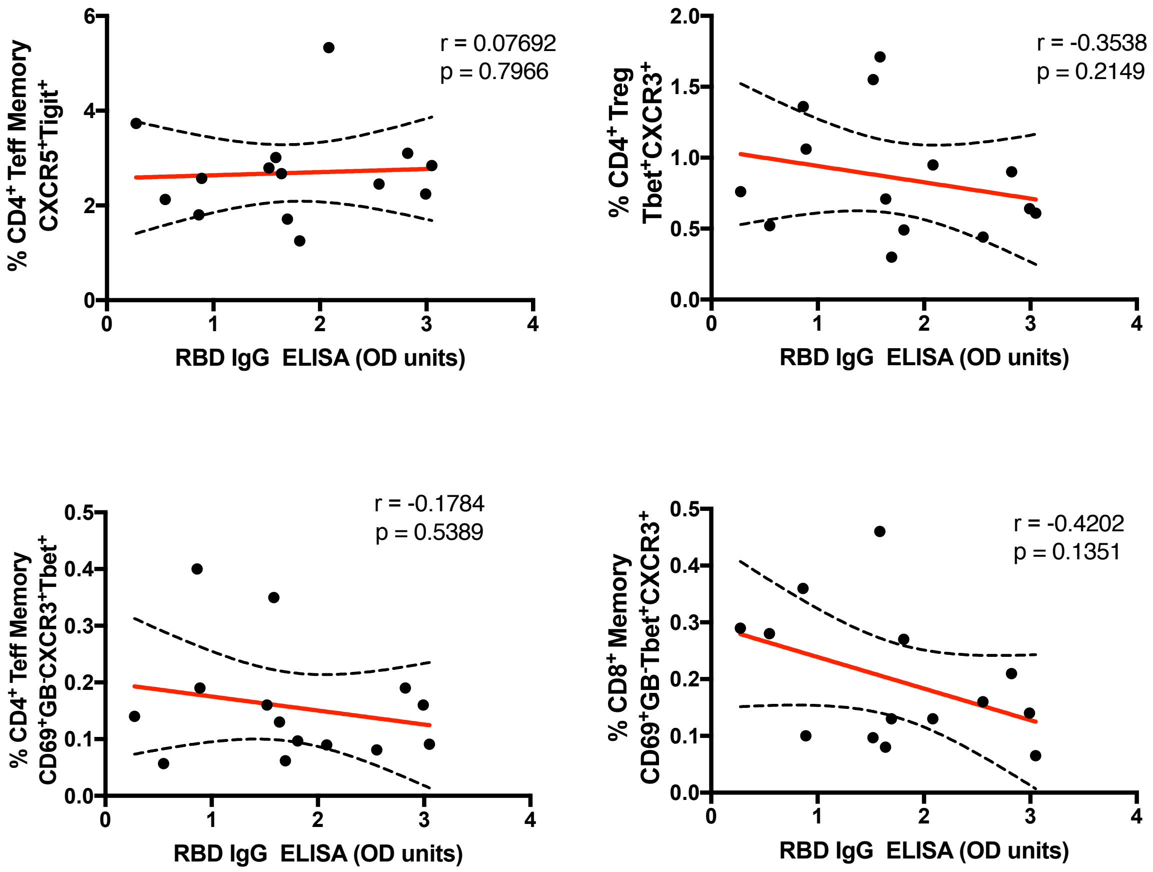


**Supplemental Figure 8.** Correlation of $CD4^{+}CD25^{+/-}FoxP3^{-}(T_{eff})CD45RO^{+}CD45RA^{-}CXCR5^{+}TIGIT^{+}$, $CD3^{+}CD4^{+}CD25^{+}FoxP3^{+}(T_{reg})CXCR3^{+}Tbet^{+}$, $CD3^{+}CD4^{+}CD25^{+/-}FoxP3^{-}(T_{eff})CD45RO^{+}CD45RA^{-}GB^{-}CD69^{+}Tbet^{+}CXCR3^{+}$ and $CD8^{+}CD45RO^{+}CD45RA^{-}GB^{-}CD69^{+}Tbet^{+}CXCR3^{+}$ cell subsets with anti-RBD IgG OD (optical density) readings. Cell frequencies expressed as percentages of $CD4^{+}$ or $CD8^{+}$ parent lineages. Red solid line: line of best fit, black dashed line: 95% confidence interval and r: Spearman correlation.

| Panel A | | | | | | | |
|---|---|---|---|---|---|---|---|
| **Metal** | **Antibody** | **Isotype** | **Clone** | **Company** | **Catalogue Number** | **Lot Number** | **Concentration (µg/ml)** |
| **89** | **Barcode A** | mIgG1 | HI30 | Fluidigm | 30890003B | 2671906 | 7.5 |
| **106** | **Barcode B** | mIgG1 | HI30 | Biolegend | 304002 | B263746 | 6 |
| **110** | **CD19** | mIgG1 | HIB19 | Biolegend | 302202 | B286428 | 6 |
| **111** | **CD45RA** | mIgG2b | HI100 | Biolegend | 304102 | B255475 | 6 |
| **112** | **CD14** | mIgG2a | TüK4 | Invitrogen | MA5-16955 | UL2902165A | 6 |
| **113** | **Barcode C** | mIgG1 | HI30 | Biolegend | 304002 | B263746 | 6 |
| **114** | **CD56** | mIgG2b | NCAM16.2 | BD Pharmingen | 559043 | 9113714 | 6 |
| **115** | **Barcode D** | mIgG1 | HI30 | Biolegend | 304002 | B263746 | 7 |
| **116** | **CD8** | mIgG1 | SK1 | Biolegend | 344702 | B249147 | 5 |
| **139** | **HLA-DR** | mIgG2a | L243 | Biolegend | 307602 | B268800 | 6 |
| **141** | **CD95 *** | mIgG1 | DX2 | Biolegend | 305602 | B241963 | 5 |
| **142** | **CD45RO** | mIgG2a | UCHL1 | Biolegend | 304202 | B232536 | 5 |
| **143** | **CD3** | mIgG1 | UCHT1 | Biolegend | 300402 | B242090 | 6 |
| **144** | **IL6 *** | Rat IgG1 | MQ2-14A5 | Biolegend | 501110 | B206535 | 3 |
| **145** | **IL4** | mIgG1 | 8D4-8 | Biolegend | 500702 | B178232 | 4 |
| **146** | **Anti-FITC (TCRγδ)** | mIgG1 | FIT-22 | Biolegend | 408302 | B236716 | 6 |
| **147** | **PD-1** | mIgG1 | EH12.2H7 | Biolegend | 329941 | B237504 | 8 |
| **148** | **CD4** | mIgG1 | SK3 | Biolegend | 344602 | B249168 | 6 |
| **149** | **CD127** | mIgG1 | A019D5 | Biolegend | 351302 | B269887 | 6 |
| **150** | **IL-22** | mIgG1 | 22URTI | Fluidigm | 3150007B | 2131718 | 6 |
| **151** | **GATA3** | Rat IgG2b | TWAJ | eBioscience | 14-9966-82 | 4271873 | 5 |
| **152** | **TNF-a** | mIgG1 | Mab11 | Biolegend | 502902 | B223054 | 3 |
| **153** | **CD25** | mIgG1 | 2A3 | BD Pharmingen | 347640 | 3353776 | 5 |
| **154** | **CD7** | mIgG2a | CD7-6B7 | Biolegend | 343102 | B207245 | 5 |
| **155** | **CD152** | mIgG2a | BNI3 | BD Pharmingen | 550405 | 6021911 | 4 |
| **156** | **CD28** | mIgG1 | CD28.2 | Biolegend | 302902 | B229977 | 4 |
| **157** | **CXCR5** | Rat IgG2b | RF8B2 | BD | 552032 | 7026764 | 5 |
| **158** | **CCR7** | mIgG2a | G043H7 | Biolegend | 353202 | B248230 | 5 |
| **159** | **CXCR3 *** | mIgG1 | G025H7 | Biolegend | 353718 | B201589 | 7 |
| **160** | **CD161** | mIgG1 | HP-3G10 | Biolegend | 339902 | B260197 | 6 |
| **161** | **IRF7 *** | mIgG2b | 12G9A36 | Biolegend | 656002 | B252631 | 5 |
| **162** | **RORγt** | Rat IgG2a | AFKJS-9 | eBioscience | 14-6988-82 | 4310958 | 3 |
| **163** | **TBX21/T-bet** | mIgG1 | 4B10 | BioXell | BE0100 | 396810612 | 5 |
| **164** | **Anti-PE (TCRαβ)** | mIgG1 | PE001 | Biolegend | 408102 | B220677 | 8 |
| **165** | **FoxP3** | mIgG1 | 236A/E7 | eBioscience | 14-4777-82 | 4339062 | 7 |
| **166** | **Ki67** | mIgG1 | 20Raj1 | eBioscience | 14-5699-82 | 4297961 | 4 |
| **167** | **TCRVα7.2** | mIgG1 | 3C10 | Biolegend | 351702 | B209777 | 7 |
| **168** | **IFN-γ** | mIgG1 | B27 | Biolegend | 506502 | B195505 | 4 |
| ***169*** | **IL-17A** | mIgG1 | BL168 | Biolegend | 512302 | B186379 | 4 |
| **170** | **IL-8** | mIgG2b | BH0814 | Biolegend | 514602 | B269392 | 5 |
| **171** | **TIGIT *** | mIgG1 | MBSA43 | Invitrogen | 16-9500-82 | 2114058 | 5 |
| **172** | **CD31** | mIgG1 | WM59 | Biolegend | 303102 | B220532 | 5 |
| **173** | **GranzymeB** | mIgG1 | CLB-GB11 | Abcam | ab103159 | GR3207987-2 | 4 |
| **174** | **CD69** | mIgG1 | FN50 | Biolegend | 310902 | B220113 | 5 |
| **175** | **IL10** | Rat IgG2a, κ | JES3-19F1 | Biolegend | 506802 | B286824 | 3 |
| **176** | **iCOS *** | Armenian Hamster IgG | C398.4A | Biolegend | 313502 | B183127 | 5 |
| **209** | **CD16** | mIgG1 | 3G8 | Fluidigm | 3209002B | 2381907 | 6 |

**Supplemental Table 1.** Antibodies used for mass cytometry (COVID-19 Panel A). The antibodies' clones, source, catalogue numbers, concentrations and mass cytometry channels used for their detection are listed here. Barcodes A, B, C and D are the different lanthanide metal conjugated anti-human CD45 antibodies used for the barcoding of different PBMC samples prior to their staining with antibodies directed against cell surface antigens. *marks the antibodies that were added to the original EPIC Panel A to repurpose the panel and make it more relevant to the COVID-19 pandemic.

| Panel B | | | | | | | |
|---|---|---|---|---|---|---|---|
| **Metal** | **Antibody** | **Isotype** | **Clone** | **Company** | **Catalogue Number** | **Lot Number** | **Concentration (µg/ml)** |
| 89 | **Barcode A** | mIgG1 | HI30 | Fluidigm | 30890003B | 2671906 | 7.5 |
| 106 | **Barcode B** | mIgG1 | HI30 | Biolegend | 304002 | B263746 | 6 |
| 110 | **CD19** | mIgG1 | HIB19 | Biolegend | 302202 | B286428 | 6 |
| 111 | **CD45RA *** | mIgG2b | HI100 | Biolegend | 304102 | B255475 | 6 |
| 112 | **CD14** | mIgG2a | TüK4 | Invitrogen | MA5-16955 | UL2902165A | 6 |
| 113 | **Barcode C** | mIgG1 | HI30 | Biolegend | 304002 | B263746 | 6 |
| 114 | **CD56** | mIgG2b | NCAM16.2 | BD Pharmingen | 559043 | 9113714 | 6 |
| 115 | **Barcode D** | mIgG1 | HI30 | Biolegend | 304002 | B263746 | 7 |
| 116 | **CD8** | mIgG1 | SK1 | Biolegend | 344702 | B249147 | 5 |
| 139 | **HLA-DR** | mIgG2a | L243 | Biolegend | 307602 | B217166 | 6 |
| 141 | **CD95 *** | mIgG1 | DX2 | Biolegend | 305602 | B241963 | 5 |
| 142 | **CD11B** | mIgG1 | ICRF44 | Biolegend | 301302 | B199213 | 6 |
| 143 | **CD3** | mIgG1 | UCHT1 | Biolegend | 300402 | B242090 | 6 |
| 144 | **ACE-2 *** | mIgG1 | 171608 | R&D System | MAB9331 | UAL0420031 | 5 |
| 145 | **CD10** | mIgG1 | HI10a | Biolegend | 312202 | B229509 | 6 |
| 146 | **IgD** | mIgG2a | Ia6-2 | Biolegend | 348202 | B167341 | 6 |
| 147 | **CD1C** | mIgG1 | L161 | Biolegend | 331502 | B216381 | 7 |
| 148 | **CD4** | mIgG1 | SK3 | Biolegend | 344602 | B249168 | 6 |
| 149 | **CD21** | mIgG1 | Bu32 | Biolegend | 354902 | B203785 | 5 |
| 150 | **Anti-biotin (SARS-CoV-2 spike) *** | mIgG2a | 1D4-C5 | Biolegend | 409002 | B176326 | 5 |
| 151 | **CXCR4** | mIgG2a | 12G5 | Biolegend | 306502 | B217390 | 5 |
| 152 | **NKp46** | mIgG1 | 9E2 | Biolegend | 331902 | B180373 | 5 |
| 153 | **CD303** | mIgG2a | 201A | Biolegend | 354202 | B201480 | 6 |
| 154 | **CD27** | mIgG1 | O323 | Biolegend | 302802 | B227356 | 6 |
| 155 | **IgG** | Mouse $IgG_1$ | G18-145 | BD Pharmingen | 555784 | 5234968 | 4 |
| 156 | **PD-L1** | mIgG2b | 29E.2A3 | Biolegend | 329702 | B203790 | 6 |
| 157 | **CD11c** | mIgG1 | Bu15 | Biolegend | 337202 | B201002 | 6 |
| 158 | **CD141** | mIgG1 | M80 | Biolegend | 344102 | B257252 | 6 |
| 159 | **CXCR3 *** | mIgG1 | G025H7 | Biolegend | 353718 | B201589 | 10 |
| 160 | **IgM** | mIgG1 | MHM-88 | Biolegend | 314502 | B201822 | 5 |
| 161 | **IRF7 *** | mIgG2b | 12G9A36 | Biolegend | 656002 | B252631 | 5 |
| 162 | **CD62L** | mIgG1 | DREG-56 | Biolegend | 304835 | B213271 | 5 |
| 163 | **CD38** | mIgG1 | HIT2 | Biolegend | 303502 | B213310 | 5 |
| 164 | **CX3CR1** | mIgG1 | K0124E1 | Biolegend | 355702 | B247766 | 12 |
| 165 | **Anti-biotin (SARS-CoV-2 spike) *** | mIgG2a | 1D4C5 | Fluidigm | 3165012B | 1151301 | 5 |
| 166 | **CD24** | Mouse IgG2a | ML5 | Biolegend | 311102 | B206874 | 6 |
| 167 | **CD80** | mIgG1 | 2D10 | Biolegend | 305202 | B194733 | 5 |
| 168 | **IgA** | mIgG1 | G18-1 | BD Pharmingen | 555886 | 4009539 | 4 |
| 169 | **CD304** | Rat IgG2a | 12C2 | Biolegend | 354502 | B213314 | 6 |
| 170 | **NKG2D** | mIgG1 | 1D11 | Biolegend | 320802 | B228271 | 5 |
| 171 | **CD23 (FcεRII)** | mIgG1 | EBVCS-5 | Biolegend | 338502 | B201559 | 6 |
| 172 | **CD86** | Mouse IgG2b | IT2.2 | Biolegend | 305402 | B194735 | 6 |
| 173 | **Granzyme B** | mIgG1 | CLB-GB11 | Abcam | ab103159 | GR3207987-2 | 4 |
| 174 | **CD40** | Mouse IgG1 | 5C3 | Biolegend | 334325 | B201401 | 5 |
| 175 | **IL10** | Rat IgG2a, κ | JES3-19F1 | Biolegend | 506802 | B286824 | 3 |
| 176 | **CD138 *** | mIgG1 | DL101 | Biolegend | 353211 | B223016 | 5 |
| 209 | **CD16** | mIgG1 | 3G8 | Fluidigm | 3209002B | 2381907 | 6 |

**Supplemental Table 2.** Antibodies used for mass cytometry (COVID-19 Panel B). The antibodies' clones, source, catalogue numbers, concentrations and mass cytometry channels used for their detection are listed here. Barcodes A, B, C and D are the metal conjugated anti-human CD45 antibodies used for the barcoding of different PBMC samples prior to their staining with antibodies directed against cell surface antigens. *marks the antibodies that were added to the original EPIC Panel B to repurpose the panel and make it more relevant for the COVID-19 pandemic.

| Characteristics | COVID-19 (n = 19) | HD (n = 9) |
|---|---|---|
| **Age, median (IQR, range) years** | 10 (7 to 15, 3 to 36) | 9 (5 to 15.5, 2 to 39)# |
| **Gender, n (%)** | | |
| Female | 10 (52.6%) | 3 (33.3%) |
| Male | 9 (47.4%) | 6 (66.7%) |
| **Ethnicity, n (%)** | | |
| Malay | 10 (52.6%) | 5 (55.6%) |
| Chinese | 4 (21.1%) | 3 (33.3%) |
| Others | 5 (26.3%) | 1 (11.1%) |
| Asymptomatic infection | 4 | N.A. |
| Symptomatic infection | 15 | N.A. |
| Required supplemental oxygen or mechanical ventilation | 0 | N.A. |

**Supplemental Table 3.** Clinical characteristics of COVID-19 patients. Convalescent samples collection: median: 48 (IQR: 35 to 61) days from symptom onset or first nasopharyngeal swab positive. Age-matched controls were used experimentally with no significant difference in ages between the COVID-19 patients and healthy donors (HD), # p = 0.6032 (Mann-Whitney U (two-tailed) test. IQR: interquartile range, N.A.: not applicable.

**A**

| No. | Peptide start | Peptide end | Length | Sequence | HLA restriction | Core | Score | Rank | Endotoxin level | Working concentration per reaction (µg/mL) |
|---|---|---|---|---|---|---|---|---|---|---|
| 1 | 339 | 347 | 9 | GEVFNATRF | HLA-B*40:01<br>HLA-B*44:02<br>HLA*B-44:03 | n.a. | n.a. | n.a. | ≤0.01 EU/ug | 10 |
| 2 | 408 | 417 | 10 | RQIAPGQTGK | HLA-A*03:01 | RQIAPGQTGK | 0.895652 | 0.03 | ≤0.01 EU/ug | 20 |
| 3 | 417 | 425 | 9 | KIADYNYKL | HLA-A*02:01 | KIADYNYKL | 0.908998 | 0.04 | ≤0.01 EU/ug | 10 |
| 4 | 526 | 534 | 9 | GPKKSTNLV | HLA-B*07:02<br>HLA-B*08:01 | n.a. | n.a. | n.a. | ≤0.01 EU/ug | 5 |

**B**

| No. | Peptide start | Peptide end | Length | Sequence | HLA restriction | Median consensus profile | Endotoxin level | Working concentration per reaction (µg/mL) |
|---|---|---|---|---|---|---|---|---|
| 1 | 316 | 350 | 15 | SNFRVQPTESIVRFP | MHC Class II | 16 | ≤0.01 EU/ug | 10 |
| 2 | 451 | 465 | 15 | YLYRLFRKSNLKPFE | MHC Class II | 9.2 | ≤0.01 EU/ug | 10 |
| 3 | 461 | 475 | 15 | LKPFERDISTEIYQA | MHC Class II | 14 | ≤0.01 EU/ug | 10 |

**Supplemental Table 4.** Predicted T cell epitopes from SARS-CoV-2 spike glycoprotein (1). **(A)** Predicted MHC class I-restricted epitopes based on the NetMHCpan EL 4.0. (2), whereby "Core" refers to the minimal 9 amino acid binding core directly in contact with the MHC, "Score" refers to the raw prediction score, and "Rank" refers to the predicted affinity compared to a set of random natural peptides with strong binders having a percentage rank of less than 0.5. **(B)** Predicted MHC class II-restricted epitopes based on Tepitool (3), whereby "Median consensus profile" refers to the median IEDB consensus percentile ranks predicted for a set of 7 most frequently occurring MHC class II alleles. n.a. = non-applicable, EU= endotoxin activity.

**Supplemental References**

1. Grifoni A, Sidney J, Zhang Y, Scheuermann RH, Peters B, Sette A. A Sequence Homology and Bioinformatic Approach Can Predict Candidate Targets for Immune Responses to SARS-CoV-2. *Cell host & microbe* (2020) 27(4):671-80.e2. Epub 2020/03/19. doi: 10.1016/j.chom.2020.03.002. PubMed PMID: 32183941; PubMed Central PMCID: PMCPMC7142693.
2. Jurtz V, Paul S, Andreatta M, Marcatili P, Peters B, Nielsen M. NetMHCpan-4.0: Improved Peptide-MHC Class I Interaction Predictions Integrating Eluted Ligand and Peptide Binding Affinity Data. *Journal of immunology (Baltimore, Md : 1950)* (2017) 199(9):3360-8. Epub 2017/10/06. doi: 10.4049/jimmunol.1700893. PubMed PMID: 28978689; PubMed Central PMCID: PMCPMC5679736.
3. Paul S, Sidney J, Sette A, Peters B. TepiTool: A Pipeline for Computational Prediction of T Cell Epitope Candidates. *Current protocols in immunology* (2016) 114:18.9.1-.9.24. Epub 2016/08/02. doi: 10.1002/cpim.12. PubMed PMID: 27479659; PubMed Central PMCID: PMCPMC4981331.
